# Old wine into new wineskins? “Legacy data” in research on Roman Period East Germanic iron smelting

**DOI:** 10.1371/journal.pone.0289771

**Published:** 2023-10-19

**Authors:** Grzegorz Żabiński, Jarosław Gramacki, Artur Gramacki, Ivan S. Stepanov, Marcin Woźniak

**Affiliations:** 1 Institute of History, Jan Długosz University in Częstochowa, Częstochowa, PL; 2 Computer Center, University of Zielona Góra, Zielona Góra, PL; 3 Institute of Control and Computation Engineering, University of Zielona Góra, Zielona Góra, PL; 4 Curt-Engelhorn-Zentrum Archäometrie gGmbH, Mannheim, DE; 5 The Stefan Woyda Museum of Ancient Masovian Metallurgy, Pruszków, PL; German Archaeological Institute: Deutsches Archaologisches Institut, GERMANY

## Abstract

This paper discusses the use of “legacy data” in research on Roman Period iron smelting in the territory of the Przeworsk Culture in Magna Germania. The dataset includes results of 240 analyses of smelting slag and iron ores chemistry. A majority of these analyses were conducted in the 1950s-1980s. The quality of these data is far below present-day standards. Only major elements were reported, analytical methods were often not specified (although optical emission spectroscopy and wet chemical analyses can be assumed in such cases) and information on detection limits, precision and accuracy of the results is missing. In spite of this, a Principal Component Analysis-Agglomerative Hierarchical Clustering treatment successfully isolated observations from the three main iron smelting regions of the Przeworsk Culture (the Holy Cross Mountains, Masovia and Silesia). These results to a degree confirm a theory proposed in the 1960s by J. Piaskowski, according to whom it was possible to distinguish iron produced in the Holy Cross Mountains from the iron produced elsewhere in the territory of what is now Poland on the basis of metal characteristics. These findings will pave the way to the ongoing research project on the Przeworsk Culture metallurgy. It is also argued that, apart from a search for new methods in iron provenance studies, more attention should be paid to results of earlier analyses and to a compilation of legacy databases. The other result is an open and flexible Agglomerative Hierarchical Clustering R code to examine discrimination between production areas and to propose artefact provenance patterns in a convenient interactive way using the R development environment.

## Introduction

**Fig 1:**
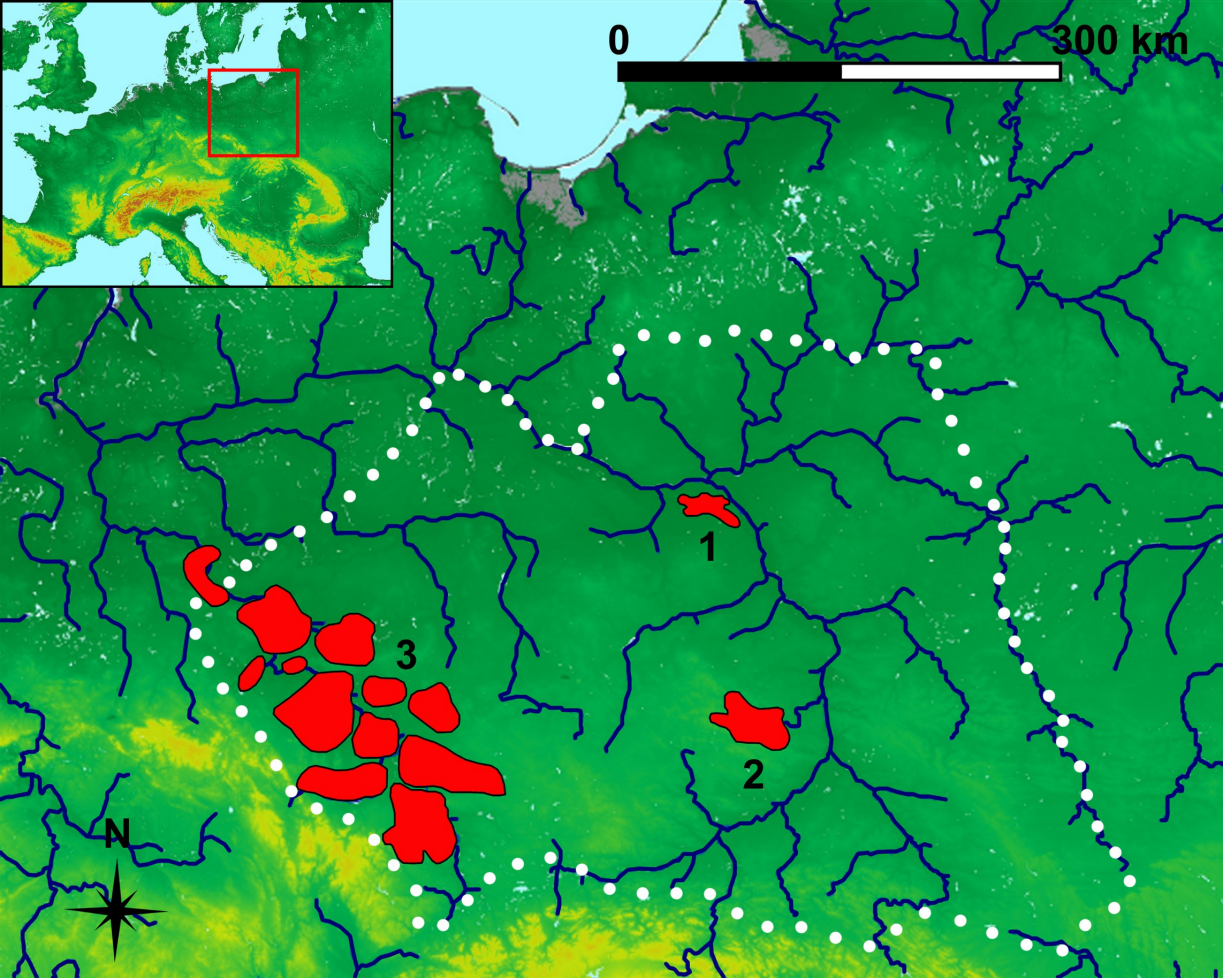
Przeworsk Culture’s ironmaking regions. Regions’ extent and the Przeworsk Culture territory in the 1^st^-mid-2^nd^ c. AD (black dots) after [16]. Background map: *We acknowledge the use of imagery provided by services from NASA’s Global Imagery Browse Services (GIBS)*, *part of NASA’s Earth Observing System Data and Information System (EOSDIS)*. River network was re-drawn by G. Żabiński after FAO AQUASTAT, https://data.apps.fao.org/aquamaps/ Accessed 7 May 2023. This afterdrawing is similar but not identical to the original image and is therefore for illustrative purposes only. 1 –Masovia; 2 –Holy Cross Mountains; 3 –Silesian regions.

This paper discusses results of 240 analyses of the chemistry of smelting slag and iron ores from the territory of the Przeworsk Culture. This culture was perhaps related to Vandal tribes and existed between the 2^nd^ c. BC and the mid-5^th^ c. AD in the eastern part of Magna Germania. The core area of this culture was the southern and central part of what is now Poland [1–10]. This culture is known for a wide use of iron and a developed iron metallurgy. Its two main iron smelting regions, i.e., the Holy Cross Mountains and Masovia, were the largest ironmaking centres in non-Roman Europe. The third significant production area was Silesia. It was, however, not one region but rather a dozen or so local clusters (Fig 1). While the Holy Cross Mountains and Masovian iron smelting may have been partially organised to serve external economic and political needs, the Silesian production was probably mainly oriented at local demand [11–17].

A majority of the discussed analyses were conducted in the 1950s-1980s, and their quality does not meet present-day standards (see below). However, it is argued that even with the use of this imperfect dataset two aims can be achieved. The first one is to at least partially verify a theory proposed in the 1960s by J. Piaskowski with regard to the metal provenance from individual iron smelting regions of this culture. The other aim is to re-test the Principal Component Analysis-Agglomerative Hierarchical Clustering (PCA-AHC) method of discrimination between individual smelting areas, so that it can be applied in new research with the use of main and trace element data obtained with the most up-to-date analytical methods.

Almost from the beginning of systematic studies on the Przeworsk Culture iron metallurgy in the 1950s, the question of metal provenance was put in the focus of scholary attention. In 1963, J. Piaskowski isolated a few groups of iron artefacts that were manufactured in the territory of what is now Poland in the 1^st^-4^th^ c. AD. One of these were artefacts made of metal that was smelted in the Holy Cross Mountains region (so-called “Holy Cross Mountains metal”). The most important characteristics of this metal were its low P content (generally in the range of 0.01–0.18%, maximally up to 0.31wt%), and a usually low (below 0.3wt% C) and uneven level of carburisation, although high-carbon steel artefacts were also reported. Intentional carburisation or forge-welding of iron and steel were presumably not known to local ironworkers. The content of P_2_O_5_ in slag was 0.12–0.58wt%. P-low hematite ores from the mine in Rudki, Kielce District, were supposedly the main raw material, which was why the metal smelted in this region was also P-low, whereas P-high ores were only limitedly used. Regarding Lower (Western) Silesia, local slag contained 0.73–7.24wt% P_2_O_5_, while the P content in Lower Silesian iron was 0.15–0.35wt%. Local artefacts were found to be poorly carburised (mainly ferritic), and local iron smelters supposedly used P-rich bog ores. Artefacts from Upper (Eastern) Silesia may have been somewhat similar to those from the Holy Cross Mountains with regard to the metal characteristics. They were unevenly carburised (below 0.3wt% C), their P content was 0.02–0.07wt%, and no carburisation or forge-welding was known there. The content of P_2_O_5_ in slag was 0.31–0.65wt%, indicating the use of P-low ores. In Masovia, P-rich bog ores were used, and local artefacts were ferritic. Their P content was 0.15–0.61 or even 0.4–1.0wt%, indicating their high brittleness. Such metal could not be carburised or quench-hardened. Masovian slag contained 2.84–10.80wt% P_2_O_5_. These characteristics of metal allowed J. Piaskowski to differentiate between the Holy Cross Mountains metal and the metal produced in other regions. The P content was considered the most effective discriminative parameter, and its upper limit of 0.08wt% was used to distinguish about 68% of Holy Cross Mountains artefacts from those made elsewhere in what is now Poland [18–20] (Table 1).

**Table 1:** The main characteristics of Przeworsk Culture iron after J. Piaskowski [1,8–20].

Region	Ores used	P (wt%) in the metal	P_2_O_5_ (wt%) in slag	C (wt%) in the metal	Artefact technology
Holy Cross Mountains	mostly P-low hematite	0.01–0.18 (max. 0.31),	0.12–0.58, avg. 0.30	uneven, up to 0.3; high-C steel possible	no intentional carburising, no forge-welding of iron and steel
		avg. 0.03			
Masovia	P-rich bog ores	0.15–0.61,	2.84–10.80, avg. 7.70	ferritic metal	no carburising possible
		avg. 0.44			
		or			
		0.4–1.0			
Lower Silesia	P-rich bog ores	0.15–0.35,	0.73–7.24,	low, mainly ferritic metal	
		avg. 0.24	avg. 2.98		
Upper Silesia	P-low ores	0.02–0.07,	0.31–0.65,	uneven, mostly below 0.3	no intentional carburising, no forge-welding of iron and steel
		avg. 0.03	avg. 0.55		

The theory of the “Holy Cross Mountains metal” provoked a heated debate. The main claims against it questioned the provenance of the studied artefacts from the Holy Cross Mountains, doubted the existence of a single manufacturing tradition in this region, or stressed the low number of analysed artefacts. Critics touched upon the discrimination based on P contents or degree of carburisation. Generally, it was argued that these characteristics were typical of bloomery iron as a material rather than of the Holy Cross Mountains metal specifically (see a discussion in [18]). However, J. Piaskowski continued to apply these criteria in subsequent studies (e.g., [21–31]). In his later works, a greater significance was given to the P content and the level of carburisation of the metal.

In 2006, Z. Kędzierski and J. Stępiński discussed results of analyses of slag and iron lumps from the Holy Cross Mountains and of some Roman Period iron artefacts, but with no examinations of slag inclusions in their metal. They objected to the use of P as a provenance criterion, saying that this element is commonly present in ores and its partition between metal and slag strongly depends on the smelting technology and later processing. According to these scholars, it was therefore impossible to clearly identify the characteristics of the “Holy Cross Mountains metal” [32]. Sz. Orzechowski was also critical about J. Piaskowski’s theory. He noted that the ores used by Holy Cross Mountains smelters were probably strongly diversified, so it would be hard to isolate particular characteristics of locally made iron as discriminating. Indeed, a whole array of ore types (hematite, siderite, spherosiderite, and limonite) occur in this region including the mine in Rudki, Kielce District, which was perhaps the only underground iron ore mine beyond the Roman Empire [11, 15; see also 12, 14].

However, while assessing J. Piaskowski’s idea, Z. Kędzierski and J. Stępiński did not consider the developments in iron provenance studies made until the early 2000s. As the newest achievements in this field made in other regions are well-known, we only briefly mention the most significant works. V. F. Buchwald and H. Wivel stated that more or less steady ratios of some major oxides could often be observed in slag inclusions in iron. These oxides are Non-Reduced Compounds (NRCs) as they do not undergo reduction in the bloomery process. Such ratios could be considered a signature of furnace charge [33–35]. Ph. Dillmann and M. L’Héritier confirmed mostly constant NRC ratios in slag inclusions throughout all manufacturing stages from blooms to semi-products, stressing the meaning of these ratios as a smelting system signature. They also proposed an identification method of smelting-related slag inclusions [36]. Alterations of the chemistry of smelting-related slag inclusions during post-smelting stages were discussed by A. Disser and co-authors [37].

M. Charlton and colleagues developed a new identification model of smelting-related slag inclusions in artefacts [38], which was modified by A. Disser and co-authors [39, see also 40, 41]. M. Charlton and colleagues also proposed a major oxide multivariate method of artefact provenancing with the use of PCA, Linear Discriminant Analysis (LDA) and Kernel Density Estimation (KDE) [38, 42]. Attention was also paid to trace elements, as major elements may better identify smelting systems than metal sources [43–45].

S. Leroy and co-authors integrated these developments. Iron sources and artefacts data were first pre-filtered with regard to their MnO and P_2_O_5_ levels. Then, LDA and AHC on major oxides and trace elements were conducted [46; for pre-filtering see also 47]. Major oxides and lithophile trace elements with PCA as a discrimination method were applied by M. L’Héritier and colleagues [48]. A. Disser and co-authors used a combined PCA-AHC treatment of major and trace elements [49, 50; see also 51–54].

Yet another research trend are attempts at coupling trace elements with Pb or Fe isotopic ratios [55–57; cf. 58]. A promising approach is the analysis of Os isotopic ratios developed by M. Brauns and co-authors [59–61]. A combination of Os isotopic ratios and major and trace elements, the latter processed by the PCA-AHC method, was applied by Ph. Dillmann and colleagues [62].

E. Bérard and colleagues compared PCA and LDA results [63, 64], or solely relied on PCA [65]. A. Jouttijärvi coupled AHC and Discriminant Analysis (DA) [66]. An approach combining the use of PCA-AHC and large databases of previously published major and trace elemental analyses (“legacy datasets”) of iron ores was successfully applied to the provenance of Early Iron Age corroded iron artefacts by I. Stepanov and co-authors [67]. M. L’Héritier and co-authors used a variety of methods such as PCA, LDA, and AHC [68]. J. Gramacki and A. Gramacki developed a multi-classifier method of provenancing of artefacts to their possible metal sources [69].

Thus, in the recent decades there has been a considerable progress in iron provenance research including the use of advanced analytical instruments which allow for studying new variables (chemistry and isotope data), and of sophisticated statistical approaches. It can be only asked why none of these have been applied so far in archaeological iron provenance studies concerning the territory of what is now Poland. This is particularly surprising for the two largest smelting regions in non-Roman Europe, and thus provides a major justification to the present work.

## Materials and methods

### Nature of the data

A research project was initiated in 2020 to examine the chemistry of slag and ore samples from the three main ironmaking regions of the Przeworsk Culture. This will allow for an identification of possible regional patterns and lay a foundation for future studies, which would also consider artefacts and isotopic variables. However, before the results of new analyses are processed, it was decided to discuss previous ore and slag chemistry data. Based on available literature [11, 18–20, 22, 27, 28, 34, 70–89], a dataset with 240 observations was compiled (S1 Dataset). Ore analyses with Fe contents below 10% and analyses of smithing slag were obviously not considered. Our verification of the theory of the “Holy Cross Mountains metal” with the use of this dataset must of course be considered tentative, as we examined no metal artefacts and all findings were made based only on the smelting slag and ore analyses.

**Fig 2:**
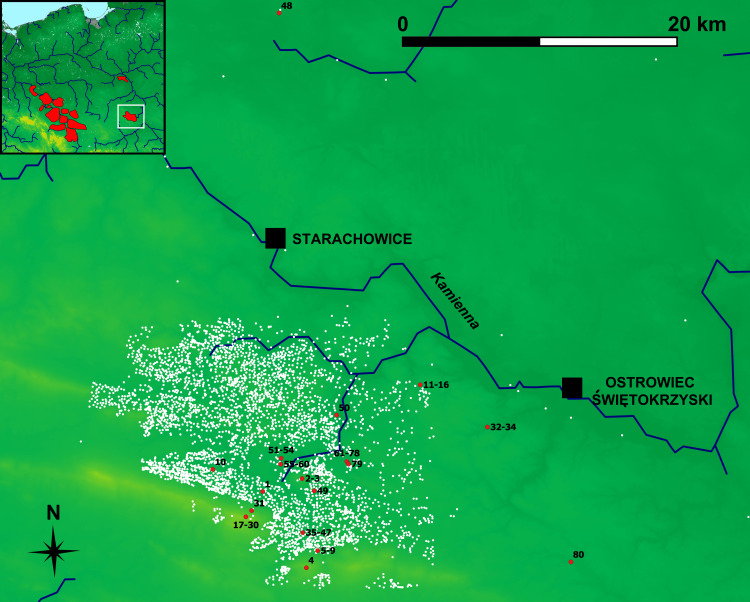
Holy Cross Mountains ironmaking region of the Przeworsk Culture. Background map: *We acknowledge the use of imagery provided by services from NASA’s Global Imagery Browse Services (GIBS)*, *part of NASA’s Earth Observing System Data and Information System (EOSDIS)*. River network was re-drawn by G. Żabiński after FAO AQUASTAT, https://data.apps.fao.org/aquamaps/ Accessed 7 May 2023. This afterdrawing is similar but not identical to the original image and is therefore for illustrative purposes only. Red points–find places of the samples discussed in the paper. White points–other ironmaking-related sites.

**Fig 3:**
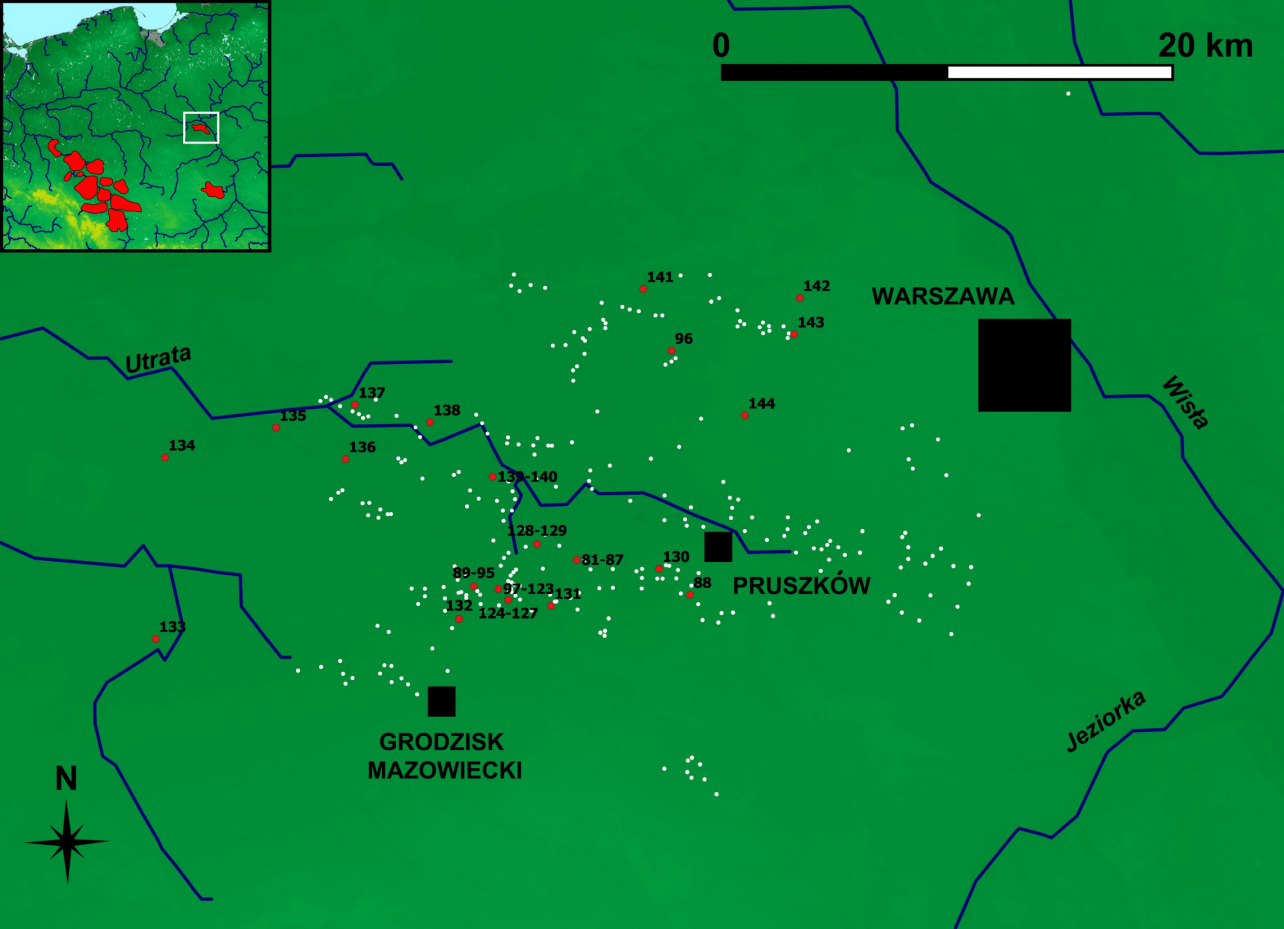
Masovian ironmaking region of the Przeworsk Culture. Background map: *We acknowledge the use of imagery provided by services from NASA’s Global Imagery Browse Services (GIBS)*, *part of NASA’s Earth Observing System Data and Information System (EOSDIS)*. River network was re-drawn by G. Żabiński after FAO AQUASTAT, https://data.apps.fao.org/aquamaps/ Accessed 7 May 2023. This afterdrawing is similar but not identical to the original image and is therefore for illustrative purposes only. Red points–find places of the samples discussed in the paper. White points–other ironmaking-related sites.

**Fig 4:**
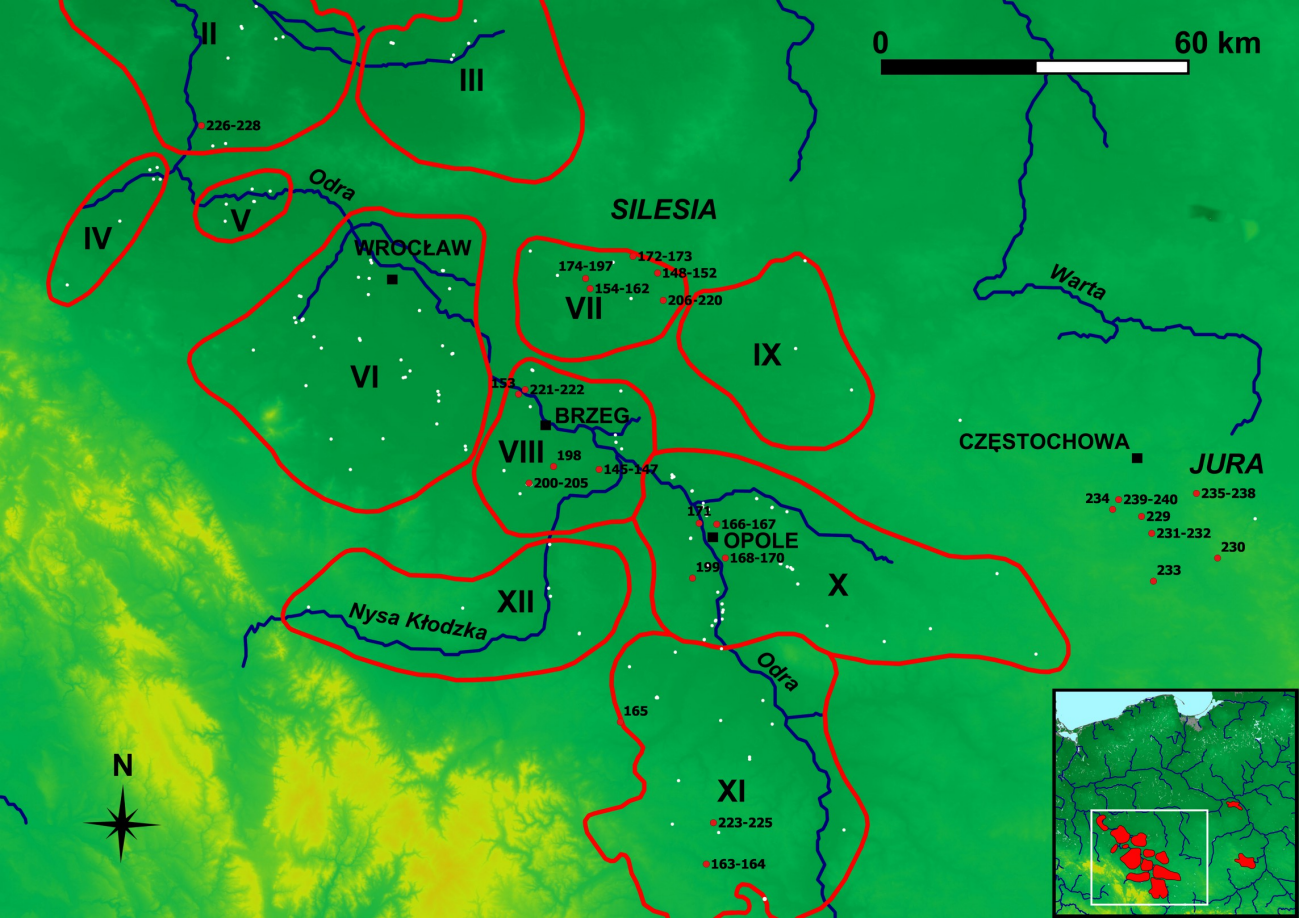
Silesian ironmaking regions of the Przeworsk Culture and the finds from the Kraków-Częstochowa Jura. Background map: *We acknowledge the use of imagery provided by services from NASA’s Global Imagery Browse Services (GIBS)*, *part of NASA’s Earth Observing System Data and Information System (EOSDIS)*. River network was re-drawn by G. Żabiński after FAO AQUASTAT, https://data.apps.fao.org/aquamaps/ Accessed 7 May 2023. This afterdrawing is similar but not identical to the original image and is therefore for illustrative purposes only. Red points–find places of the samples discussed in the paper. White points–other ironmaking-related sites. Regions–II: Rivers Dolna Barycz and Odra; III: River Górna Barycz; IV: River Kaczawa; V: Środa Śląska; VI: Rivers Bystrzyca and Oława; VII: River Widawa; VIII: Brzeg; IX: River Stobrawa; X: Strzelce Opolskie–Opole; XI: Głubczyce; XII: Nysa. Region I (Głogów) is not shown for the sake of the map’s legibility. Region borders after [11].

Our dataset includes observations from all main ironmaking regions of the Przeworsk Culture: the Holy Cross Mountains (n = 80), Masovia (n = 64) and Silesia (n = 84). There are also 12 observations from the Kraków-Częstochowa Jura (Figs 2–4). These, however, are either late medieval (slag) or present-day (ore) finds and are considered only to see how they separate from or overlap with the data from the three main regions. Data on other ironmaking-related sites in Figs 2–4 and 13 and 14 are provided after [11].

The quality of these data is far below present-day standards (S1 Dataset, Sheet “Raw Data”). Only selected main elements were reported in most cases. Analytical methods were often not specified, although optical emission spectroscopy and wet chemical analyses can be assumed in these cases (as briefly mentioned in, e.g., [25, 79, 90]). Information on detection limits, or precision and accuracy of the results is missing, and only a few more recent studies stand out [34, 78, 84], which account for 79 out of 240 observations. Some variables were given either as elements or as oxides in different studies, so all data were converted into elements for consistency. The Silesian analyses come from only 5 out of 12 local regions, which raises representativeness problems. The samples chronology is often only generally defined, although about 75% of these date to the Pre-Roman or the Roman Period. Several ore samples have no dating at all or come from present-day prospection. Thus, in most cases these samples should be considered “geological ores,” rather than “archaeological ones.” In some cases there is no reliable information about the find place. Find context data (layer, feature, etc.) are either missing or kept to minimum. Therefore, the usefulness of these data was doubted [11, 91]. However, as demonstrated below, these shortcomings did not prevent us from obtaining (archaeo)logically coherent and thus convincing results.

## Research methods

Before describing and explaining our own protocol, a more detailed analysis of methods used in some previous studies is necessary. Regarding the statistical approach, LDA as a supervised method also uses the information on the group to which observations belong, while PCA and AHC are unsupervised approaches, where this information is absent. S. Leroy and colleagues preferred LDA over PCA, assuming that the former would better link artefacts to smelting systems, while PCA would maximise the spread of smelting slag inclusion chemistry in an artefact [92]. M. L’Héritier and colleagues believed that a combination of all the named methods could produce the best results. However, such LDA options as confusion matrices of metal source observations, and prediction tables with artefact-to-source classifications were not exploited. The prediction option would not consider unknown production areas, so the analysis of LDA graphs was the only choice [68].

A. Disser and colleagues argued that LDA “requires the availability of iron products that were undoubtedly made locally” which was difficult to accomplish for every possible metal source. PCA was preferred as it analyses chemistry information independently of the archaeological context of finds. Then, AHC with Ward’s agglomeration method was used for isolating clusters of similar observations. The “elbow-type method” of clustering consisted in truncating the dendrogram at a height from which the total variance of the dataset does not strongly decrease. The analysis proceeded in three steps until a provenance of artefacts from a single production area was proposed [50].

G. Pagès and co-authors mainly used PCA, while AHC was an auxiliary method. An artefact provenance was verified based on a production area-artefact match on PC graphs gathering over 75% of the data variance [53]. E. Bérard and co-authors solely relied on PC graphs with 90% of variance [65].

It can be discussed how these findings translate into our approach selection. LDA requires high inter-class variability and low intra-class variability to produce a sensible separation. Moreover, the number of observations in each group must exceed the number of variables. In our case, there are four regions with varying numbers of observations (between 12 and 84), while only a few variables are available. The use of the information on the regional provenance of the samples would not be reasonable due to geological reasons, as various ore types occur in the Holy Cross Mountains [11, 12, 15] (see below). Such regional sub-groups are difficult to a priori determine, as different ore minerals are not distributed in geographically separable clusters [11]. If we defined our groups as find places, we would have groups with around 30 observations, and those with a few or one only. Thus, neither of the two named conditions could be met. Furthermore, although at some sites there were probably less than a dozen or so smelting operations, there were also sites with thousands of furnaces, such as Biskupice, Site 1, Pruszków District in Masovia (about 5,000). It may have operated over a period of c. 150 BC-c. 250 AD. Even 15,000 furnaces are supposed at Milanówek-Falęcin, Site 8, Grodzisk Mazowiecki and Pruszków Districts in Masovia [11–13, 15, 17, 83]. Therefore, it can hardly be expected that all ores used at such sites came from one source only.

All these premises suggest the use of unsupervised methods. However, PCA as the only or the main method is not fully reliable. Even if 90% of variance is considered, the remaining 10% can still contain valuable information on relationships between observations. Moreover, classifications solely based on visual assessments of graphs are inevitably biased by human error. Therefore, the use of a clustering algorithm is in our opinion indispensable [93].

Another issue is the use of P and Mn (or their oxides) for pre-filtering purposes, as their discriminating potential is indisputable [46, 51, 53]. P_2_O_5_ cannot be included in multivariate methods, as it is easily reducible and goes both to metal and slag [36, 38, 50, 51]. V. F. Buchwald and H. Wivel mentioned MnO among the NRCs [33]. Ph. Dillmann and M. L’Héritier remarked that it was more or less reduced in the bloomery process [36], and S. Bauvais and co-authors stated that MnO was not an NRC [51]. However, experiments demonstrated that Mn largely passes into the slag in the bloomery process. Thus, its behaviour is close to that of the NRCs [94–96]. A complication may probably be related to the fact that the Mn content is often more variable in iron ores than the contents of most other lithophile elements. This promotes a higher variation of Mn in slag [97–99]. Therefore, we included Mn into the multivariate dataset [38, 50, 62, 69], and we used P for filtering purposes only.

The next issue is data transformation method. We analyse compositional data, i.e., they sum to a constant unit (100%, if all measured variables are normalised to 100%). As individual NRC contents in ores, slag, and slag inclusions may strongly vary, they must be provided with a similar weight. Otherwise, results of multivariate treatment may be distorted by elements with the highest variance. Several methods have been discussed, e.g., [38, 69, 100], and one of these is J. Aitchison’s centered log-ratio [101]:

$$y=\log[\frac{x}{g(x_{1}\ldots x_{N})}]$$ (1)

y–transformed value

x–value of the variable in a given observation

g(x_1_ . . .x_N_)–geometrical mean of individual variables in a given observation

It was applied in many previous works [46, 50–53, 62–65, 67, 68], but it was expressed with a different equation, which produces exactly the same results:

$$X_{E}=\log([E])-\frac{1}{N}\sum_{k=1}^{N}\log([E_{k}])$$ (2)

Thus, we applied this data transformation, using the first equation, as computationally simpler.

A comment must also be made on the variable selection. Possible variables in our dataset were Mg, Al, Si, K, Ca, and Mn [38, 50, 62, 67, 69]. However, K was absent or 0 in 152 out of 240 observations which was likely related to difficulties of measurement of alkalis by early analytical methods. Mg was absent or 0 in 67 observations (S1 Dataset, Sheet “Raw Data”). Therefore, replacing so many missing contents with the lowest regional values (e.g., [38]) would produce distorted results. Yet another option, i.e., a removal of the observations with missing values (34 observations altogether, excluding those with missing K or Mg values) was not considered, due to the small size of the dataset. Thus, available variables were Al, Si, Ca and Mn only. However, we conducted variance tests to check whether all these in fact discriminate between the regions in question. The effect of the missing data replacement with the lowest regional values was tested by comparing the obtained results with those produced by datasets where the missing values were replaced by other imputation methods.

Our research protocol consists of the following steps:

1. Missing Al, Si, Ca, and Mn values are replaced with the lowest regional values. The number of the replaced values is 43 (Al– 26, Mn– 11, Si– 3, Ca– 3), i.e., less than 4.5% of all the values in the dataset (240 observations x 4 variables = 960). Then, the data undergo centered log-ratio transformation
2. ANOVA is conducted on transformed variables to study their “global” variance. ANOVA is a parametric test which requires the normal distribution of data and the homogeneity of variance in each analysed group. If these conditions cannot be met, the Kruskal-Wallis non-parametric test is used. The normality of data distributions and the homogeneity of variances are examined using the Shapiro-Wilk, the Levene and the Barlett tests respectively. The variables discriminating power in regional pairs is examined using the Tukey and Bonferroni post-hoc tests (see also S1 Dataset, Sheet “ANOVA”) and is then visualised in boxplots. P values are shown in their raw form, as P is used for filtering only
3. If not all the tested variables have a discriminant potential, the data transformation is conducted again on the raw values of the discriminating variables. If all the variables discriminate, this step is not necessary
4. A covariance-type PCA based on Pearson’s correlation coefficient index of similarity is conducted on the transformed variables
5. PC scores are processed by AHC (dissimilarity type, Euclidian distance, Ward’s method of agglomeration). The AHC treatment can be also done directly on the transformed variables. However, we included PCA as it conveniently visualises the results obtained in one of the research steps. Dendrograms are first automatically truncated based on maximum inertia. If necessary, the truncation is repeated manually using the “elbow-type method.” Observation classes are isolated based on the truncation results
6. The final classification of observations (sub-classes) is done by filtering the AHC classes according to the P level. Due to problems with exact quantification of P with earlier methods, we used a threshold of 1.0% P_2_O_5_ (~0.437% P), dividing the observations into P-low and P-high ones. If the observations are first pre-filtered and then undergo the multivariate treatment, the eventual results are very similar. However, we used P-filtering in the last stage, as pre-filtering would produce two datasets which would have to be processed separately
7. These sub-classes are discussed against the find context background of the observations (region, find place, and find type such as ore or slag) to assess the archaeological soundness of the classification

Calculations in our study were made using R Ver. 4. 3. 0. and XLSTAT Ver. 2021.5.1. All calculation details, R codes and the obtained results can be found in S1 File.

## Results and discussion

**Fig 5:**
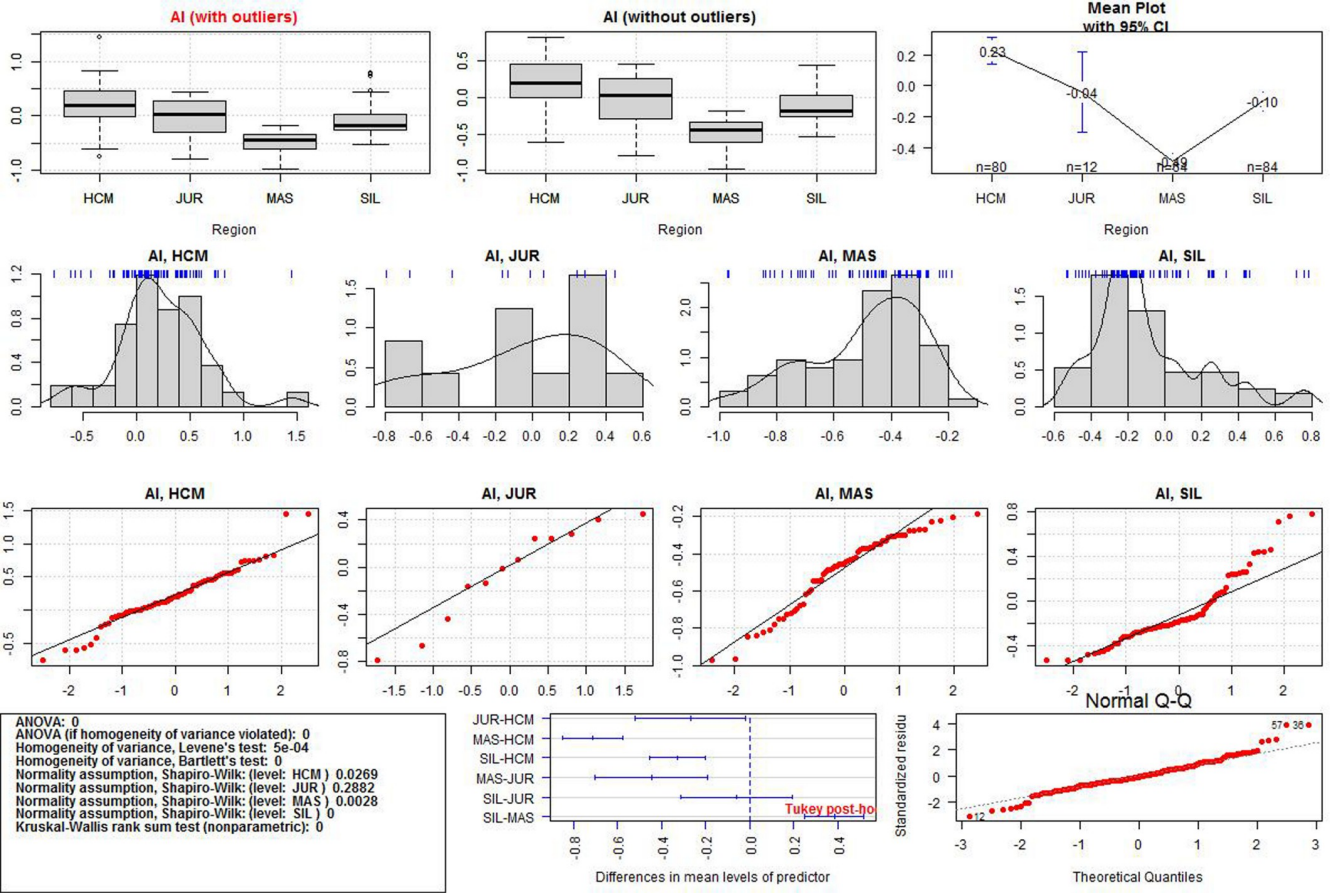
ANOVA results for Al values in the three iron smelting regions of the Przeworsk Culture and in the Kraków-Częstochowa Jura. HCM–Holy Cross Mountains; MAS–Masovia; SIL–Silesia; JUR–Jura.

**Fig 6:**
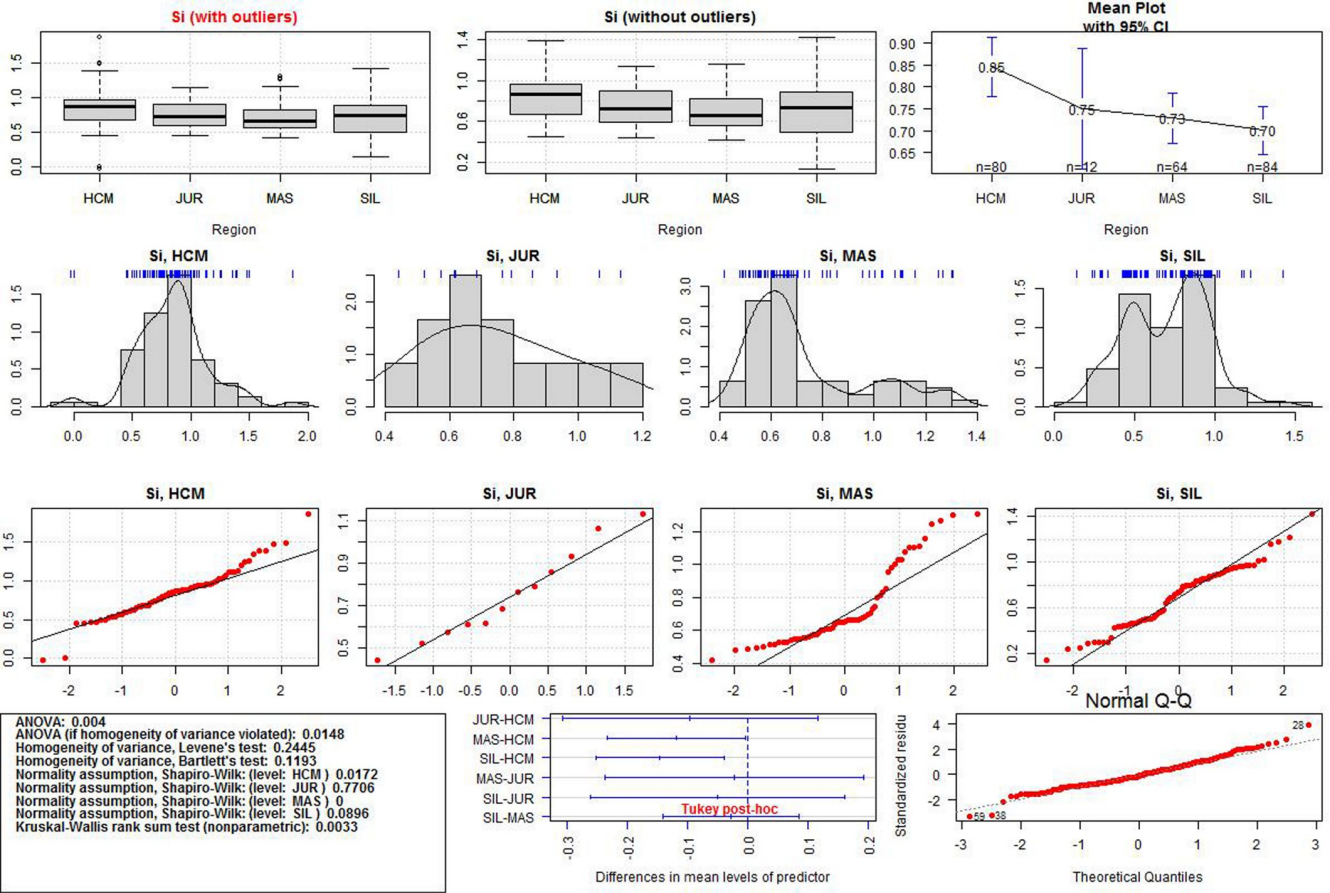
ANOVA results for Si values in the three iron smelting regions of the Przeworsk Culture and in the Kraków-Częstochowa Jura. HCM–Holy Cross Mountains; MAS–Masovia; SIL–Silesia; JUR–Jura.

**Fig 7:**
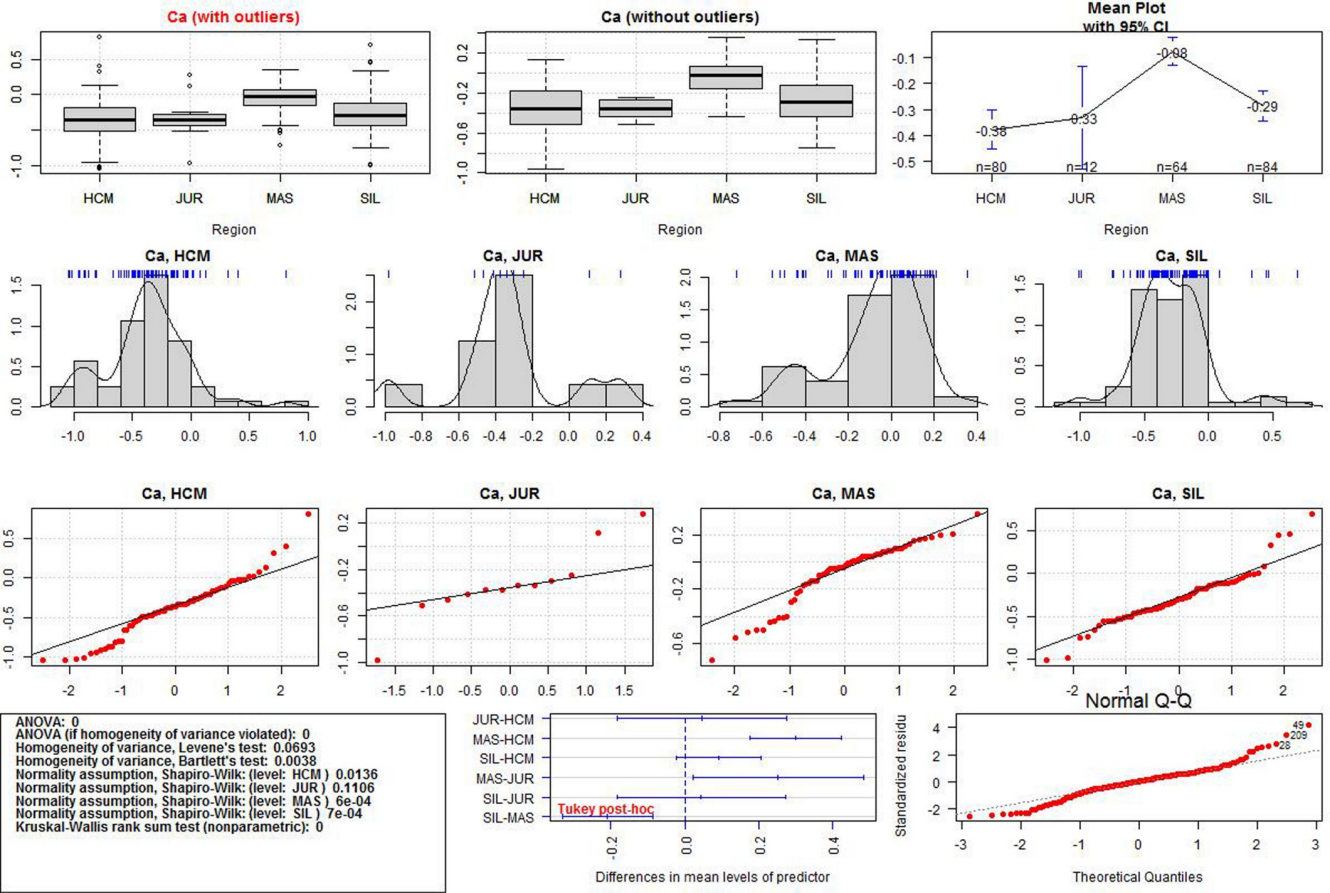
ANOVA results for Ca values in the three iron smelting regions of the Przeworsk Culture and in the Kraków-Częstochowa Jura. HCM–Holy Cross Mountains; MAS–Masovia; SIL–Silesia; JUR–Jura.

**Fig 8:**
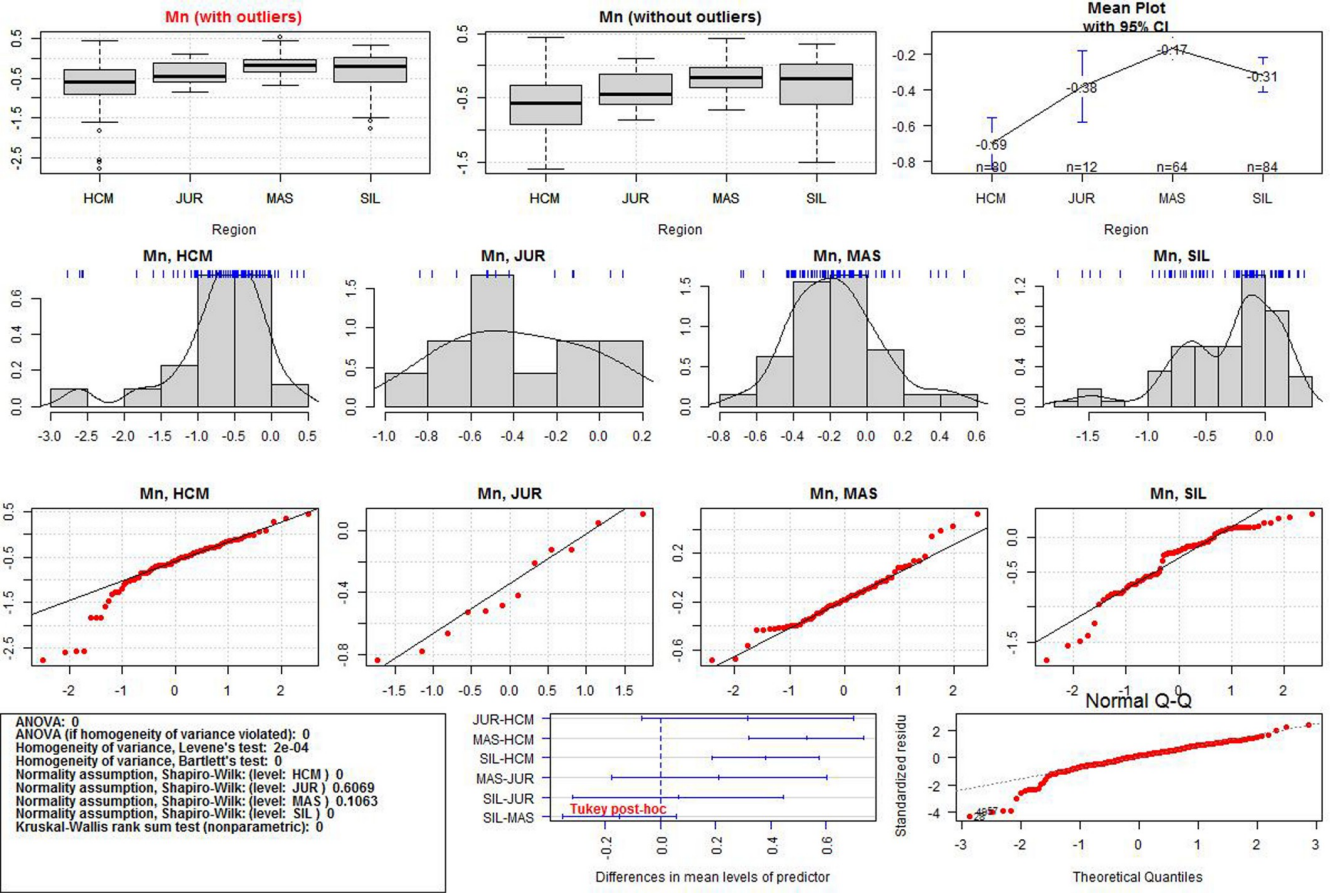
ANOVA results for Mn values in the three iron smelting regions of the Przeworsk Culture and in the Kraków-Częstochowa Jura. HCM–Holy Cross Mountains; MAS–Masovia; SIL–Silesia; JUR–Jura.

After the data transformation, the discriminant power of Al, Si, Ca, and Mn was examined. Significant differences in the regional means were assessed by one-way ANOVA. If the normality and homogeneity assumptions were violated, the Kruskal-Wallis test was used. Statistical significance was set at p-value < 0.05.The ANOVA results for each variable and additional data visualisations are presented in Figs 5–8. The first (upper) row in each of these figures shows two boxplots where the median of the data is marked. A graph of the same data with their mean values is provided in the upper right corner. The first boxplot in the upper row of each figure was generated using the complete data for each element, while the second boxplot was produced after the removal of outliers (displayed as small circles). All the calculations in the paper were conducted on the complete dataset, whereas the boxplots without outliers were only used for the sake of comparison with those containing outliers.

The second row in each figure shows the density distributions and histograms of the variables in all the regions. We also added a rough representation of the data to each plot (blue ticks). The third row in each figure contains Q-Q normality plots of the data by regions. It is a visualisation of the fulfilment of the normality assumption in individual regions.

The box in the leftmost part of the fourth row in each figure contains a summary of the numerical results (ANOVA test, normality and homogeneity tests, Kruskal-Wallis test). Verse 1 in the box shows the result (in the form of p-values) of the classical ANOVA test. If the p-value is less than 0.05, there are statistically significant differences in the regional means of a given element. However, if this result is to be statistically reliable, the data must meet the conditions of variance homogeneity and data normality. Verse 2 contains the results of a special version of the ANOVA test which can be used if only the homogeneity condition is not met. Results of the Levene and the Barlett tests for the compliance with the first condition are offered in Verses 3 and 4. This condition is met if the p-values are less than 0.05. Verses 5–6 contain the results of data normality tests in the regions. The normality condition in each region is met if the p-value is higher than 0.05. If none of these two conditions are met, the Kruskal-Wallis test is used (Verse 9). When the p-value is less than 0.05, the differences in the regional means of a given element are statistically significant (as in Verse 1).

The central plot in the fourth row of Figs 5–8 demonstrates the results of the Tukey tests. If the horizontal line for each regional pair is beyond the 0.0 vertical dashed line, the regional means of a given element are significantly different. The rightmost figure are the Q-Q normality plots for each variable in all the regions together.

**Fig 9:**
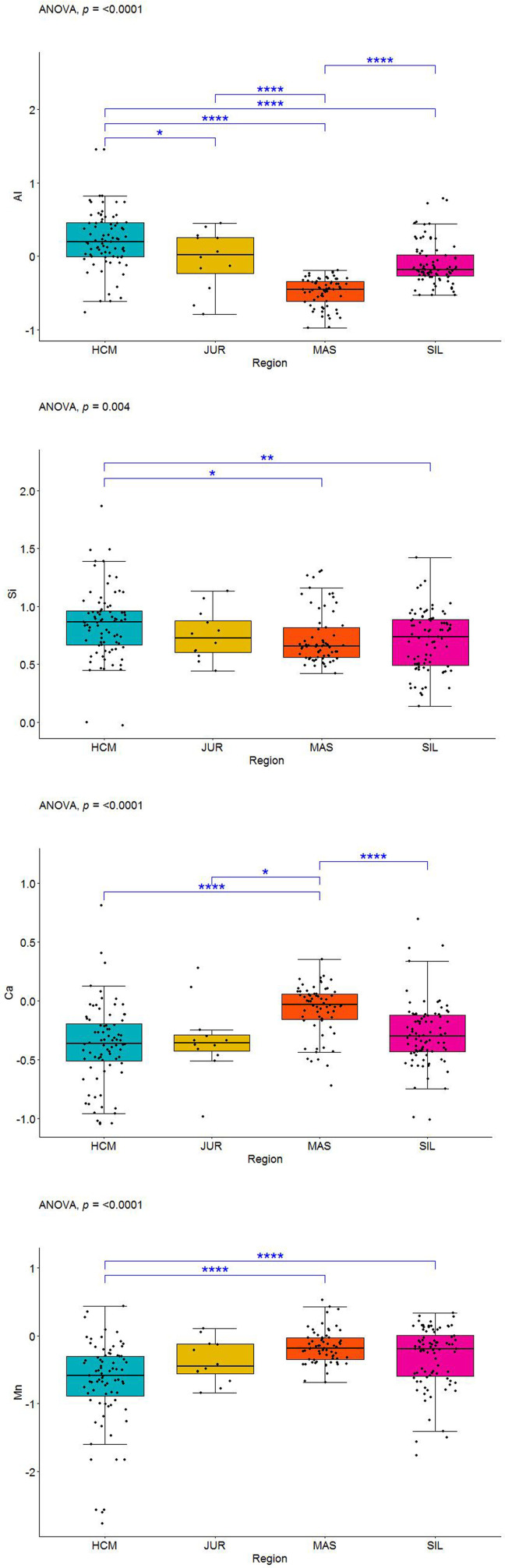
Detailed boxplots with Al, Si, Ca, and Mn values in the three iron smelting regions of the Przeworsk Culture and in the Kraków-Częstochowa Jura. HCM–Holy Cross Mountains; MAS–Masovia; SIL–Silesia; JUR–Jura. Blue asterisks indicate regional differences in the levels of each element.

Detailed boxplots for each element in the regions can be found in Fig 9. These include the Tukey tests results (blue asterisks indicate how strongly the regional values of each element vary) and visualisations of the data points (black dots). All the numerical results for all the ANOVA tests corresponding to those in Figs 5–8 can be found in S1 Text. We also include density plots of the variables for all the regions together, and separately for each region (S1 Fig).

**Fig 10:**
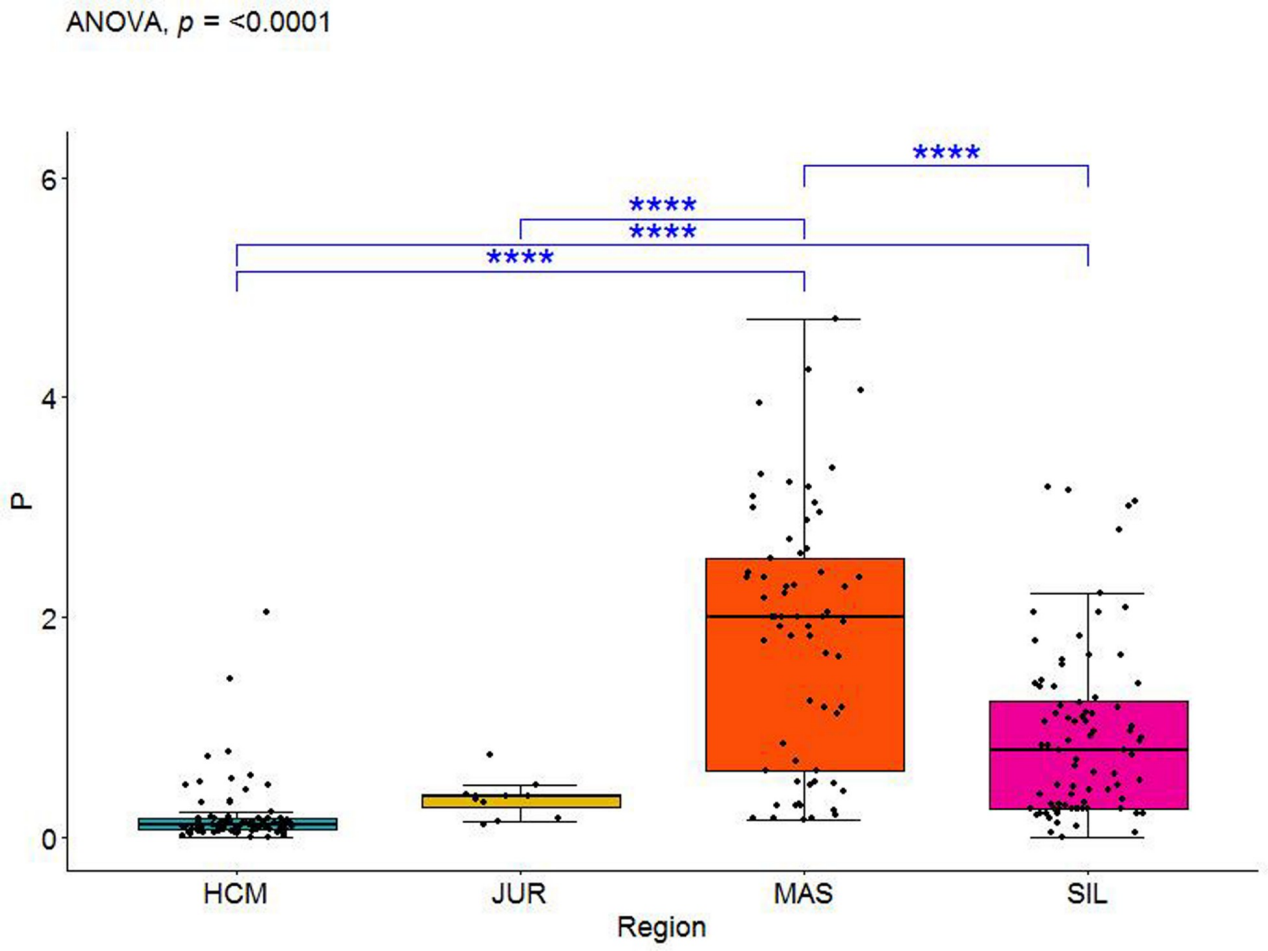
Boxplot with P raw values in the three iron smelting regions of the Przeworsk Culture and in the Kraków-Częstochowa Jura. HCM–Holy Cross Mountains; MAS–Masovia; SIL–Silesia; JUR–Jura. Blue asterisks indicate regional differences in the levels of P.

Out of possible six regional pairs, Al discriminated between five, Ca–between three, while Si and Mn between two. Thus, we retained all four elements in the multivariate dataset. A boxplot clearly proves that P has a strong discriminant potential, especially concerning the Holy Cross Mountains and Masovia (Fig 10).

**Fig 11:**
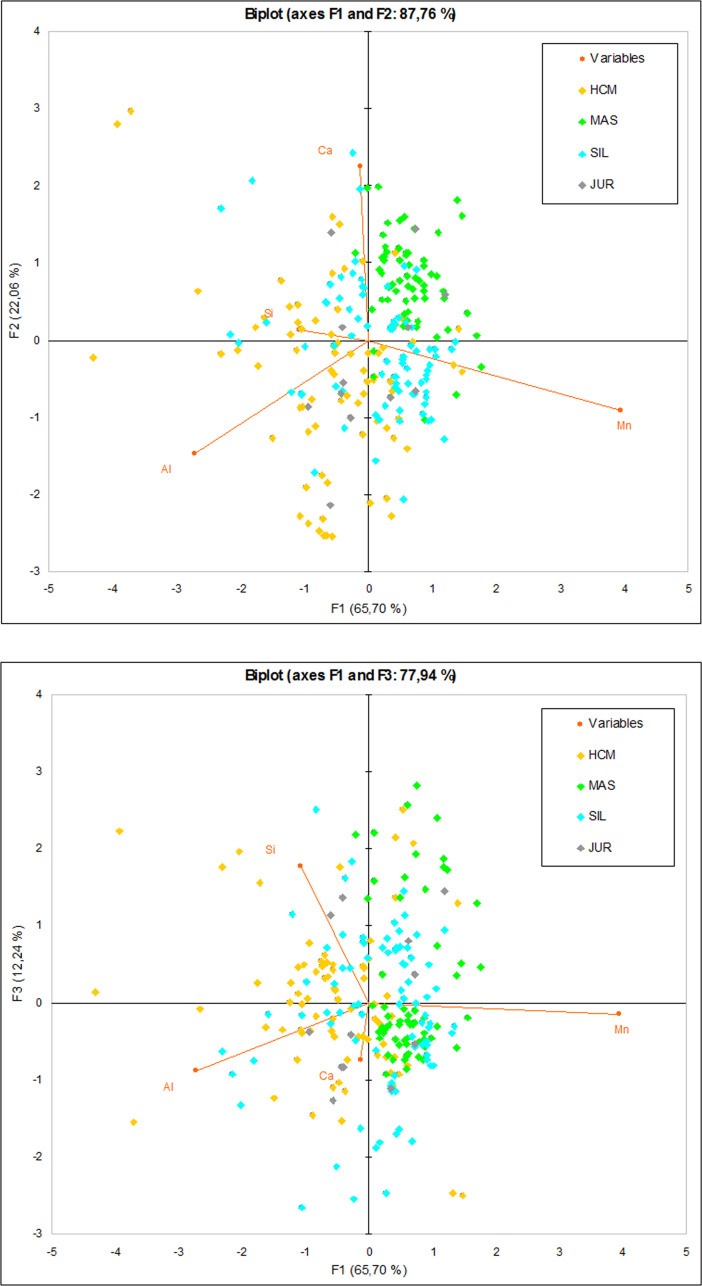
PCA graphs with the observations from the three iron smelting regions of the Przeworsk Culture and from the Kraków-Częstochowa Jura. HCM–Holy Cross Mountains; MAS–Masovia; SIL–Silesia; JUR–Jura. Variables: Al, Si, Ca, and Mn.

PCA graphs (Fig 11: PC 1 and PC 2–87.76% variability; PC 1 and PC 3–77.94% variability, PC 1–3–100% variability) demonstrate a separation between the Holy Cross Mountains and Masovia, with some in-between zone. Silesian observations overlap with these two regions, and the Silesia-Holy Cross Mountains overlap is obviously stronger. Jura observations do not form any coherent group. Concerning the relationships between the variables and the observations, there is a correlation between Al and the Holy Cross Mountains and between Ca and Masovia, which is also implied by ANOVA and the boxplots (Figs 5 and 7).

**Fig 12:**
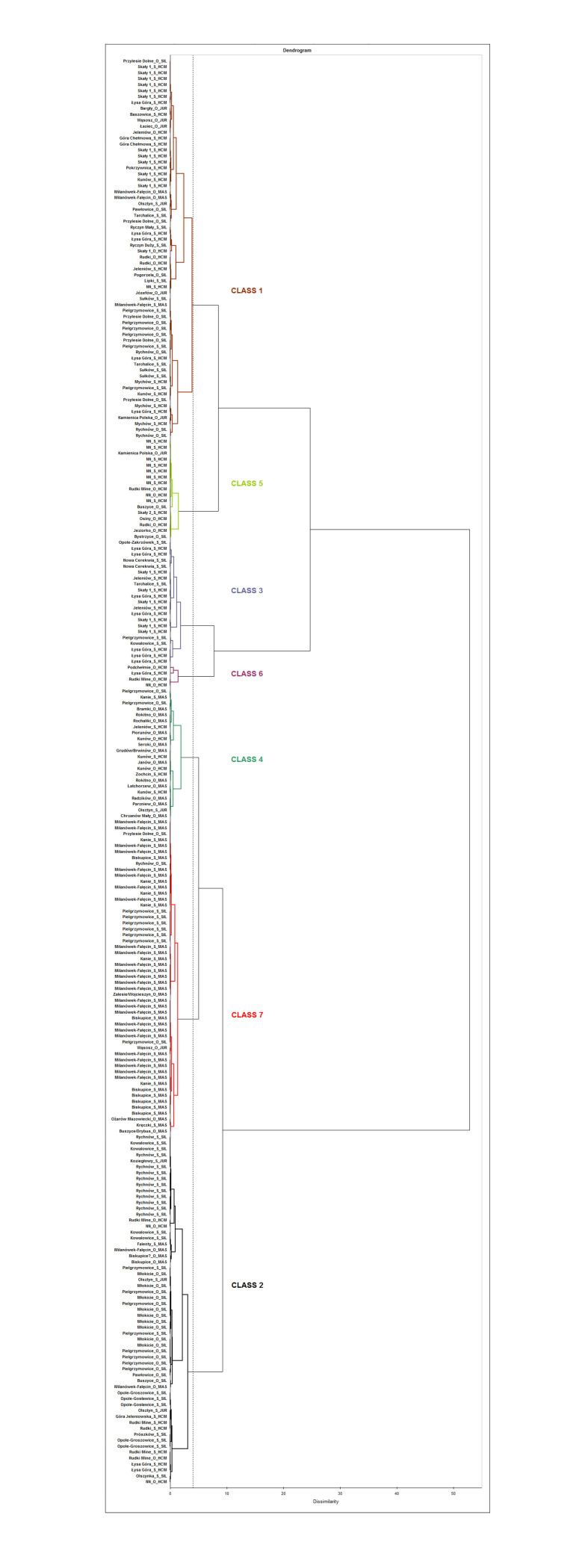
AHC dendrogram with the observations from the three iron smelting regions of the Przeworsk Culture and from the Kraków-Częstochowa Jura. HCM–Holy Cross Mountains; MAS–Masovia; SIL–Silesia; JUR–Jura. Truncation level 4.

In the next stage, the data were processed by AHC. The correctness of the clustering can be assessed by inspecting the variance decomposition for optimal classfication. The within-class variance is 27.28%, while the between-class variance is 72.72%, which obviously demonstrates that the differences within the classes are lower than those between the classes. In result of the AHC treatment, seven classes were isolated (Fig 12).

The composition of these AHC classes is the following (Table 2):

**Table 2:** AHC classes with the observations from the three iron smelting regions of the Przeworsk Culture and from the Kraków-Częstochowa Jura.

Class 1 (64)	Class 2 (59)	Class 3 (21)	Class 4 (22)	Class 5 (17)	Class 6 (4)	Class 7 (53)
HCM– 31	HCM– 10	HCM– 15	HCM– 6	HCM– 14	HCM– 4	
MAS– 3	MAS– 5		MAS– 13			MAS– 43
SIL– 24	SIL– 41	SIL– 6	SIL– 2	SIL– 2		SIL– 9
JUR– 6	JUR– 3		JUR– 1	JUR– 1		JUR– 1

HCM–Holy Cross Mountains; MAS–Masovia; SIL–Silesia; JUR–Jura.

As said above, it would be unreasonable to expect four regional classes, and almost all the obtained classes are mixed. However, Class 2 is mainly Silesian, Classes 3, 5 and 6 are either mostly or solely Holy Cross Mountains, and Class 7 is chiefly Masovian. The Jura observations are scattered in Classes 1, 2, 4, 5, and 7. A strong heterogeneity of the Holy Cross Mountains iron ores must be borne in mind here, as various minerals of different geological age such as hematite, siderite, spherosiderite, mixed argillaceous siderite-limonite and limonite occur in this region. This diversity also concerns the named mine in Rudki, Kielce District. What was reported there (apart from pyrite, which was not convenient for bloomery smelting due to its S contents) were hematite, hematite and limonite mixtures, limonite, and siderite [11, 12, 15]. Therefore, it is of no surprise that the observations from this region went into as many as six classes. Although only limonite ores are known from Silesia [11], the Silesian observations also ended up in six classes, perhaps because they come from a vast geographical area (Fig 4). The Masovian observations went into four classes, Class 7 including a majority of these (43 out of 64 Masovian observations in total). This may be caused by two reasons, namely, that the territorial extent of this smelting region is compact (Fig 3), and that only limonite bog ores are known there. However, their chemistry may strongly vary [11, 17].

For the sake of testing the data replacement effect, the PCA-AHC treatment was repeated on the datasets where the missing values were treated by three other methods. These were: Multiple Imputation by Chained Equations (MICE) with the Predictive Mean Matching (PMM) approach being the default one for numerical values; “nearest neighbour” imputation; and the removal of 34 observations with the missing values. The results obtained on the data produced by these methods varied in details, but were generally similar concerning the basic findings:

- MICE imputation for each region individually: six classes (two mostly Holy Cross Mountains, one mostly Masovian, one mostly Silesian, and two mixed)
- “nearest neighbour” imputation: six classes (three mostly Holy Cross Mountains, one mostly Masovian, one mostly Silesian, and one mixed)
- removal of the observations with the missing values: five classes (two mostly Holy Cross Mountains, one mostly Masovian, one mostly Silesian, and one mixed)

The differences that were obtained using the last dataset are perhaps mainly due to the fact that the removal affected individual regions to a various degree. The number of the removed Holy Cross Mountains observations was 11 (out of 80), Masovian– 17 (out of 64), Jura– 4 (out of 12), while merely 2 out of 84 Silesian observations were left out. Bearing all this in mind, we believe that in this case the use of the lowest regional values was the safest, albeit not a perfect option. This is because the missing values for certain elements in our dataset were in all probability mostly due to the fact that the levels of these elements were too low to be recorded with the analytical methods that were used. This strongly suggest a replacement with a low value, while the values that were proposed by different imputation methods varied considerably.

The next step involves filtering the AHC classes with the P threshold of 1.0% P_2_O_5_ (~0.437% P), so that each class is divided into two sub-classes: a P-low (A) and a P-high one (B) (Table 3).

**Table 3:** P-filtered AHC classes with the observations from the three iron smelting regions of the Przeworsk Culture and from the Kraków-Częstochowa Jura.

Obs. No.	Site_Find_Region	Region	AHC Class	P (wt %)	P-filtered Sub-Class
50	Pokrzywnica_S_HCM	HCM	1	0.0000	1A
198	Pogorzela_O_SIL	SIL	1	0.0000	1A
53	***Rudki_O_HCM***	HCM	1	0.0200	1A
54	***Rudki_O_HCM***	HCM	1	0.0200	1A
18	***Łysa Góra_S_HCM***	HCM	1	0.0432	1A
32	***Mychów_S_HCM***	HCM	1	0.0480	1A
9	***Jeleniów_S_HCM***	HCM	1	0.0611	1A
5	***Jeleniów_O_HCM***	HCM	1	0.0655	1A
78	***Skały 1_S_HCM***	HCM	1	0.0655	1A
34	***Mychów_S_HCM***	HCM	1	0.0698	1A
3	***Góra Chełmowa_S_HCM***	HCM	1	0.0786	1A
17	***Łysa Góra_S_HCM***	HCM	1	0.0794	1A
29	***Łysa Góra_S_HCM***	HCM	1	0.0960	1A
2	***Góra Chełmowa_S_HCM***	HCM	1	0.0970	1A
63	***Skały 1_S_HCM***	HCM	1	0.1004	1A
68	***Skały 1_S_HCM***	HCM	1	0.1004	1A
15	*Kunów_S_HCM*	HCM	1	0.1047	1A
33	***Mychów_S_HCM***	HCM	1	0.1047	1A
232	*Kamienica Polska_O_JUR*	JUR	1	0.1091	1A
62	***Skały 1_S_HCM***	HCM	1	0.1135	1A
71	***Skały 1_S_HCM***	HCM	1	0.1178	1A
67	***Skały 1_S_HCM***	HCM	1	0.1266	1A
61	***Skały 1_O_HCM***	HCM	1	0.1309	1A
64	***Skały 1_S_HCM***	HCM	1	0.1309	1A
65	***Skały 1_S_HCM***	HCM	1	0.1309	1A
70	***Skały 1_S_HCM***	HCM	1	0.1353	1A
66	***Skały 1_S_HCM***	HCM	1	0.1396	1A
1	Baszowice_S_HCM	HCM	1	0.1484	1A
43	NN_S_HCM	HCM	1	0.1658	1A
237	*Olsztyn_S_JUR*	JUR	1	0.1658	1A
69	***Skały 1_S_HCM***	HCM	1	0.2357	1A
225	*Sułków_S_SIL*	SIL	1	0.3142	1A
27	***Łysa Góra_S_HCM***	HCM	1	0.3273	1A
234	Łaziec_O_JUR	JUR	1	0.3491	1A
229	Bargły_O_JUR	JUR	1	0.3709	1A
240	*Wąsosz_O_JUR*	JUR	1	0.3797	1A
230	Józefów_O_JUR	JUR	1	0.4713	1B
223	***Sułków_S_SIL***	SIL	1	0.5237	1B
224	***Sułków_S_SIL***	SIL	1	0.5804	1B
172	*Pawłowice_O_SIL*	SIL	1	0.6546	1B
16	*Kunów_S_HCM*	HCM	1	0.7332	1B
25	*Łysa Góra_S_HCM*	HCM	1	0.7768	1B
204	***Przylesie Dolne_O_SIL***	SIL	1	0.8335	1B
127	***Milanówek-Falęcin_O_MAS***	MAS	1	0.8510	1B
205	***Przylesie Dolne_O_SIL***	SIL	1	0.9077	1B
203	***Przylesie Dolne_O_SIL***	SIL	1	1.0823	1B
115	***Milanówek-Falęcin_S_MAS***	MAS	1	1.1172	1B
201	***Przylesie Dolne_O_SIL***	SIL	1	1.1303	1B
125	***Milanówek-Falęcin_O_MAS***	MAS	1	1.1783	1B
153	Lipki_S_SIL	SIL	1	1.2001	1B
182	***Pielgrzymowice_S_SIL***	SIL	1	1.2656	1B
226	***Tarchalice_S_SIL***	SIL	1	1.3616	1B
227	***Tarchalice_S_SIL***	SIL	1	1.3659	1B
202	***Przylesie Dolne_O_SIL***	SIL	1	1.4314	1B
209	***Rychnów_O_SIL***	SIL	1	1.5710	1B
176	***Pielgrzymowice_O_SIL***	SIL	1	1.6147	1B
174	***Pielgrzymowice_O_SIL***	SIL	1	1.6583	1B
222	Ryczyn Mały_S_SIL	SIL	1	1.7849	1B
175	***Pielgrzymowice_O_SIL***	SIL	1	1.8329	1B
207	***Rychnów_O_SIL***	SIL	1	2.0511	1B
188	***Pielgrzymowice_S_SIL***	SIL	1	2.0947	1B
189	***Pielgrzymowice_S_SIL***	SIL	1	2.7930	1B
208	***Rychnów_O_SIL***	SIL	1	3.0112	1B
221	Ryczyn Duży_S_SIL	SIL	1	3.1595	1B
38	NN_O_HCM	HCM	2	0.0218	2A
59	***Rudki Mine_O_HCM***	HCM	2	0.0218	2A
146	*Buszyce_O_SIL*	SIL	2	0.0480	2A
37	NN_O_HCM	HCM	2	0.0524	2A
58	***Rudki Mine_O_HCM***	HCM	2	0.0524	2A
31	***Łysa Góra_S_HCM***	HCM	2	0.0600	2A
60	***Rudki Mine_S_HCM***	HCM	2	0.0829	2A
199	Prószków_S_SIL	SIL	2	0.1004	2A
170	***Opole-Groszowice_S_SIL***	SIL	2	0.1353	2A
4	Góra Jeleniowska_S_HCM	HCM	2	0.1500	2A
30	***Łysa Góra_S_HCM***	HCM	2	0.1571	2A
55	***Rudki Mine_S_HCM***	HCM	2	0.1700	2A
52	***Rudki_S_HCM***	HCM	2	0.1789	2A
168	***Opole-Groszowice_S_SIL***	SIL	2	0.1789	2A
169	***Opole-Groszowice_S_SIL***	SIL	2	0.2051	2A
151	***Kowalowice_S_SIL***	SIL	2	0.2182	2A
166	***Opole-Gosławice_S_SIL***	SIL	2	0.2182	2A
215	***Rychnów_S_SIL***	SIL	2	0.2182	2A
150	***Kowalowice_S_SIL***	SIL	2	0.2618	2A
210	***Rychnów_S_SIL***	SIL	2	0.2618	2A
211	***Rychnów_S_SIL***	SIL	2	0.2618	2A
213	***Rychnów_S_SIL***	SIL	2	0.2618	2A
216	***Rychnów_S_SIL***	SIL	2	0.2618	2A
217	***Rychnów_S_SIL***	SIL	2	0.2618	2A
218	***Rychnów_S_SIL***	SIL	2	0.2618	2A
167	***Opole-Gosławice_S_SIL***	SIL	2	0.2837	2A
214	***Rychnów_S_SIL***	SIL	2	0.3055	2A
233	Koziegłowy_S_JUR	JUR	2	0.3098	2A
212	***Rychnów_S_SIL***	SIL	2	0.3491	2A
235	***Olsztyn_S_JUR***	JUR	2	0.3709	2A
238	***Olsztyn_S_JUR***	JUR	2	0.3709	2A
220	***Rychnów_S_SIL***	SIL	2	0.3928	2A
165	Olszynka_S_SIL	SIL	2	0.4277	2A
219	*Rychnów_S_SIL*	SIL	2	0.4800	2B
124	***Milanówek-Falęcin_O_MAS***	MAS	2	0.4844	2B
129	***Biskupice*?*_O_MAS***	MAS	2	0.6110	2B
128	***Biskupice_O_MAS***	MAS	2	0.6982	2B
162	***Młokicie_O_SIL***	SIL	2	0.7419	2B
161	***Młokicie_O_SIL***	SIL	2	0.7855	2B
193	***Pielgrzymowice_O_SIL***	SIL	2	0.7855	2B
197	***Pielgrzymowice_O_SIL***	SIL	2	0.8292	2B
194	***Pielgrzymowice_O_SIL***	SIL	2	0.8728	2B
196	***Pielgrzymowice_O_SIL***	SIL	2	0.8728	2B
195	***Pielgrzymowice_O_SIL***	SIL	2	0.9164	2B
157	***Młokicie_O_SIL***	SIL	2	0.9601	2B
160	***Młokicie_O_SIL***	SIL	2	0.9601	2B
155	***Młokicie_O_SIL***	SIL	2	1.0037	2B
158	***Młokicie_O_SIL***	SIL	2	1.0474	2B
184	***Pielgrzymowice_S_SIL***	SIL	2	1.0474	2B
192	***Pielgrzymowice_O_SIL***	SIL	2	1.0474	2B
156	***Młokicie_O_SIL***	SIL	2	1.0910	2B
173	*Pawłowice_O_SIL*	SIL	2	1.1346	2B
159	***Młokicie_O_SIL***	SIL	2	1.1783	2B
88	Falenty_S_MAS	MAS	2	1.2394	2B
154	***Młokicie_O_SIL***	SIL	2	1.3965	2B
126	***Milanówek-Falęcin_O_MAS***	MAS	2	1.6496	2B
183	***Pielgrzymowice_S_SIL***	SIL	2	2.2256	2B
149	***Kowalowice_S_SIL***	SIL	2	3.0548	2B
148	***Kowalowice_S_SIL***	SIL	2	3.1857	2B
6	***Jeleniów_S_HCM***	HCM	3	0.0864	3A
75	***Skały 1_S_HCM***	HCM	3	0.0960	3A
20	***Łysa Góra_S_HCM***	HCM	3	0.1091	3A
24	***Łysa Góra_S_HCM***	HCM	3	0.1113	3A
76	***Skały 1_S_HCM***	HCM	3	0.1178	3A
74	***Skały 1_S_HCM***	HCM	3	0.1266	3A
73	***Skały 1_S_HCM***	HCM	3	0.1353	3A
8	***Jeleniów_S_HCM***	HCM	3	0.1484	3A
26	***Łysa Góra_S_HCM***	HCM	3	0.1545	3A
72	***Skały 1_S_HCM***	HCM	3	0.1746	3A
19	***Łysa Góra_S_HCM***	HCM	3	0.1920	3A
152	*Kowalowice_S_SIL*	SIL	3	0.2182	3A
77	***Skały 1_S_HCM***	HCM	3	0.3229	3A
180	*Pielgrzymowice_S_SIL*	SIL	3	0.3928	3A
163	*Nowa Cerekwia_S_SIL*	SIL	3	0.4320	3A
171	Opole-Zakrzówek_S_SIL	SIL	3	0.4582	3B
164	*Nowa Cerekwia_S_SIL*	SIL	3	0.4800	3B
23	***Łysa Góra_S_HCM***	HCM	3	0.5106	3B
21	***Łysa Góra_S_HCM***	HCM	3	0.5368	3B
228	*Tarchalice_S_SIL*	SIL	3	0.7026	3B
22	***Łysa Góra_S_HCM***	HCM	3	2.0511	3B
13	*Kunów_S_HCM*	HCM	4	0.0480	4A
7	*Jeleniów_S_HCM*	HCM	4	0.0698	4A
136	Piorunów_O_MAS	MAS	4	0.1527	4A
132	Chrzanów Mały_O_MAS	MAS	4	0.1746	4A
134	Seroki_O_MAS	MAS	4	0.1746	4A
138	Radzików_O_MAS	MAS	4	0.1746	4A
137	Rochaliki_O_MAS	MAS	4	0.1964	4A
140	***Rokitno_O_MAS***	MAS	4	0.2400	4A
131	Grudów/Brwinów_O_MAS	MAS	4	0.2837	4A
139	***Rokitno_O_MAS***	MAS	4	0.2837	4A
135	Bramki_O_MAS	MAS	4	0.3055	4A
130	Parzniew_O_MAS	MAS	4	0.4146	4A
80	Zochcin_S_HCM	HCM	4	0.4364	4A
11	***Kunów_O_HCM***	HCM	4	0.4800	4B
12	***Kunów_S_HCM***	HCM	4	0.4800	4B
143	Latchorzew_O_MAS	MAS	4	0.5019	4B
14	***Kunów_O_HCM***	HCM	4	0.5586	4B
142	Janów_O_MAS	MAS	4	0.6110	4B
236	*Olsztyn_S_JUR*	JUR	4	0.7419	4B
179	***Pielgrzymowice_O_SIL***	SIL	4	1.3965	4B
177	***Pielgrzymowice_O_SIL***	SIL	4	1.6583	4B
95	*Kanie_S_MAS*	MAS	4	2.3566	4B
51	***Rudki_O_HCM***	HCM	5	0.0000	5A
10	Jeziorko_O_HCM	HCM	5	0.0393	5A
145	*Buszyce_O_SIL*	SIL	5	0.0480	5A
79	*Skały 2_S_HCM*	HCM	5	0.0567	5A
35	NN_O_HCM	HCM	5	0.0611	5A
56	***Rudki Mine_O_HCM***	HCM	5	0.0611	5A
47	NN_S_HCM	HCM	5	0.0786	5A
45	NN_S_HCM	HCM	5	0.0829	5A
44	NN_S_HCM	HCM	5	0.0873	5A
46	NN_S_HCM	HCM	5	0.0873	5A
41	NN_S_HCM	HCM	5	0.1135	5A
231	*Kamienica Polska_O_JUR*	JUR	5	0.1484	5A
40	NN_S_HCM	HCM	5	0.1527	5A
39	NN_S_HCM	HCM	5	0.1571	5A
42	NN_S_HCM	HCM	5	0.1658	5A
48	Osiny_O_HCM	HCM	5	0.1877	5A
147	Bystrzyce_O_SIL	SIL	5	0.5848	5B
36	NN_O_HCM	HCM	6	0.0393	6A
57	*Rudki Mine_O_HCM*	HCM	6	0.0393	6A
28	*Łysa Góra_S_HCM*	HCM	6	0.3221	6A
49	Podchełmie_O_HCM	HCM	6	1.4445	6B
187	***Pielgrzymowice_S_SIL***	SIL	7	0.2182	7A
191	***Pielgrzymowice_S_SIL***	SIL	7	0.2182	7A
181	***Pielgrzymowice_S_SIL***	SIL	7	0.2618	7A
190	***Pielgrzymowice_S_SIL***	SIL	7	0.2618	7A
133	Buszyce/Drybus_O_MAS	MAS	7	0.2837	7A
185	***Pielgrzymowice_S_SIL***	SIL	7	0.3055	7A
186	***Pielgrzymowice_S_SIL***	SIL	7	0.3055	7A
239	*Wąsosz_O_JUR*	JUR	7	0.3884	7A
141	Zalesie/Wojcieszyn_O_MAS	MAS	7	0.4800	7B
144	Ożarów Mazowiecki_O_MAS	MAS	7	0.5019	7B
200	*Przylesie Dolne_O_SIL*	SIL	7	1.1172	7B
110	***Milanówek-Falęcin_S_MAS***	MAS	7	1.1783	7B
178	*Pielgrzymowice_O_SIL*	SIL	7	1.2219	7B
120	***Milanówek-Falęcin_S_MAS***	MAS	7	1.6670	7B
109	***Milanówek-Falęcin_S_MAS***	MAS	7	1.7892	7B
111	***Milanówek-Falęcin_S_MAS***	MAS	7	1.8329	7B
112	***Milanówek-Falęcin_S_MAS***	MAS	7	1.8329	7B
87	***Biskupice_S_MAS***	MAS	7	1.9202	7B
119	***Milanówek-Falęcin_S_MAS***	MAS	7	1.9202	7B
108	***Milanówek-Falęcin_S_MAS***	MAS	7	1.9638	7B
84	***Biskupice_S_MAS***	MAS	7	2.0074	7B
85	***Biskupice_S_MAS***	MAS	7	2.0074	7B
103	***Milanówek-Falęcin_S_MAS***	MAS	7	2.0074	7B
106	***Milanówek-Falęcin_S_MAS***	MAS	7	2.0074	7B
113	***Milanówek-Falęcin_S_MAS***	MAS	7	2.0074	7B
107	***Milanówek-Falęcin_S_MAS***	MAS	7	2.0511	7B
206	*Rychnów_O_SIL*	SIL	7	2.0511	7B
97	***Milanówek-Falęcin_S_MAS***	MAS	7	2.1820	7B
81	***Biskupice_S_MAS***	MAS	7	2.2169	7B
86	***Biskupice_S_MAS***	MAS	7	2.2693	7B
114	***Milanówek-Falęcin_S_MAS***	MAS	7	2.2693	7B
82	***Biskupice_S_MAS***	MAS	7	2.2867	7B
102	***Milanówek-Falęcin_S_MAS***	MAS	7	2.3566	7B
118	***Milanówek-Falęcin_S_MAS***	MAS	7	2.3697	7B
99	***Milanówek-Falęcin_S_MAS***	MAS	7	2.4002	7B
100	***Milanówek-Falęcin_S_MAS***	MAS	7	2.4002	7B
122	***Milanówek-Falęcin_S_MAS***	MAS	7	2.5311	7B
104	***Milanówek-Falęcin_S_MAS***	MAS	7	2.5748	7B
123	***Milanówek-Falęcin_S_MAS***	MAS	7	2.6184	7B
83	***Biskupice_S_MAS***	MAS	7	2.7057	7B
121	***Milanówek-Falęcin_S_MAS***	MAS	7	2.8802	7B
116	***Milanówek-Falęcin_S_MAS***	MAS	7	2.9588	7B
117	***Milanówek-Falęcin_S_MAS***	MAS	7	2.9981	7B
93	***Kanie_S_MAS***	MAS	7	3.0330	7B
98	***Milanówek-Falęcin_S_MAS***	MAS	7	3.0984	7B
105	***Milanówek-Falęcin_S_MAS***	MAS	7	3.1857	7B
101	***Milanówek-Falęcin_S_MAS***	MAS	7	3.2294	7B
96	Kręczki_S_MAS	MAS	7	3.2948	7B
89	***Kanie_S_MAS***	MAS	7	3.3603	7B
92	***Kanie_S_MAS***	MAS	7	3.9451	7B
90	***Kanie_S_MAS***	MAS	7	4.0629	7B
91	***Kanie_S_MAS***	MAS	7	4.2505	7B
94	***Kanie_S_MAS***	MAS	7	4.7131	7B

HCM–Holy Cross Mountains; MAS–Masovia; SIL–Silesia; JUR–Jura. *Cursive ‐ localities/sites with multiple samples*. ***Cursive bold–finds of Ore (O) OR Slag (S) from the same locality/site in the same sub-class***. ***Cursive bold underlined–finds of Ore (O) AND Slag (S) from the same locality/site in the same sub-class*.**

### Geography of the isolated sub-classes

As many as 11 out of 14 sub-classes are either “clean,” i.e., they contain the observations (both ore and slag) from one region only, or “almost clean,” being dominated by the observations from solely one region. These are:

- 1A: mostly Holy Cross Mountains (29 out of 36 observations)
- 1B: mostly Silesian (22 out of 28 observations)
- 2B: mostly Silesian (21 out of26 observations)
- 3A: mostly Holy Cross Mountains (12 out of 15 observations)
- 4A: mostly Masovian (10 out of 13 observations)
- 5A: mostly Holy Cross Mountains (14 out of 16 observations)
- 5B: only Silesian (1 observation)
- 6A: only Holy Cross Mountains (3 observations)
- 6B: only Holy Cross Mountains (1 observation)
- 7A: mostly Silesian (6 out of 8 observations)
- 7B: mostly Masovian (42 out of 45 observations)

Out of 240 observations, 161 (67.08%) went to such sub-classes. The relevant figures for individual regions are the following:

- Holy Cross Mountains: 59 out of 80 observations (73.75%)
- Masovia: 52 out of 64 observations (81.25%)
- Silesia: 50 out of 84 observations (59.52%)
- Jura: 0 out of 12 observations (0%)

This result demonstrates that our dataset bears a strong discriminant potential (save Jura). 2/3 of all the observations and around 3/4 of those from the Holy Cross Mountains and Masovia went to the “clean” or “almost clean” sub-classes. The discrimination for Silesia is worse, which can be partially explained by differences in the chemistries of ores and other components of smelting operations in a vast region.

Yet another issue is the clustering of observations from the same sites or localities, especially when both ore and slag data are available. It is unlikely that all smelting operations at a given site were conducted with the same components, especially at sites which operated for several centuries. Moreover, it is unknown to what extent the ore finds were representative of the smelted ores. Therefore, a 100% slag-ore match is improbable. Out of 240 observations, there were 193 multiple observations from the same sites or localities, and 110 cases with both ore and slag data. The number of the observations from the same sites or localities in the same sub-class is as many as 164 or 84.97% (193 cases– 100%). A slag-ore match occurred in 28 cases, i.e., 25.45% of all the cases with ore and slag data. Varying patterns emerge when we focus on sites or localities from which slag and ore data are more numerous. This is discussed on selected examples from Table 3.

Ten samples (seven ore and three slag) were available from Rudki, Kielce District in the Holy Cross Mountains. Six come from the mine (Site 2), while four were reported from the locality of Rudki. Ore samples belong to various minerals: hematite, hematite with siderite, and limonite (S1 Dataset, Sheet “Working Data”). Five observations (three slag and two ore) went to Sub-Class 2A, two to Sub-Class 5A (ores only), another two (ores only) to Sub-Class 1A, while one (ore) to Sub-Class 6A. Sub-Classes 1A, 5A and 6A are either mostly or solely Holy Cross Mountains, and all four sub-classes are P-low. Regarding the ore classification, both samples from Sub-Class 2A (Obs. 58 and 59) are different ore types (hematite plus siderite, and limonite respectively), while the remaining are hematite (Sub-Class 1A –Obs. 53 and 54; Sub-Class 5A –Obs. 51 and 56; Sub-Class 6A –Obs. 57). There is no simple dependency between the sub-class and the ore type, but the very facts that the Rudki ores went to different sub-classes and an ore-slag match in one of these imply the archaeological soundness of our result.

Six samples (two ore and four slag finds) come from Kunów, Site 1, Ostrowiec Świętokrzyski District in the Holy Cross Mountains. Three (only slag) went to three different sub-classes (Obs. 15 –Sub-Class 1A; Obs. 16 –Sub-Class 1B, Obs. 13 –Sub-Class 4A) while another three (two ore and one slag) ended up in Sub-Class 4B (Obs. 11, 12, and 14). Out of these sub-classes, only Sub-Class 1A is mostly Holy Cross Mountains.

Milanówek-Falęcin, Site 8, Grodzisk Mazowiecki and Pruszków Districts in Masovia was one of the largest (c. 15,000 furnaces) and longest-operating (c. 150 BC-c. AD 150) Przeworsk Culture iron smelting sites [11–13, 15, 17, 83]. Therefore, a possible diversity of the sources of smelting system components should manifest in the classification of individual samples to different sub-classes. There are 31 samples from Milanówek-Falęcin (27 slag from Site 8 and four ore from the area of these localities), although the slag samples come from three slag blocks only (S1 Dataset, Sheet “Working Data”). This locality yielded nearly a half of all the Masovian samples. 26 slag samples went to the mostly Masovian Sub-Class 7B indicating a similar provenance or smelting system. The remaining one slag sample and all ore samples ended up in the mostly Silesian Sub-Classes 1B (Obs. 115 –slag; Obs. 125 and 127 –ore) and 2B (Obs. 124 and 126 –ore).

In Silesia, 24 samples are known from Pielgrzymowice, Namysłów District (12 ore and 12 slag, S1 Dataset, Sheet “Working Data”). Two ore samples were classified to Sub-Class 4B, while six slag samples went to Sub-Class 7A (mostly Silesian). On the other hand, the number of both ore and slag observations in the same sub-classes was as many as 14. Six went to Sub-Class 1B (Obs. 174–176 –ore; Obs. 182, 188, and 189 –slag), while eight–to Sub-Class 2B (Obs. 192–197 –ore; Obs. 183–184 –slag). Both these sub-classes are mostly Silesian. The remaining samples were classified into separate Sub-Classes 3A (Obs. 180 –slag) and 7B (Obs. 178 –ore). The case of Pielgrzymowice (analogous to Rudki) demonstrates that ores from one and the same locality may display different chemistries.

An examination of how this classification translates into the sites’ geography can also be done by a scrutiny of some of the “clean” or “almost clean” sub-classes. Sub-Class 1A (36 observations) gathers 29 Holy Cross Mountains samples (Fig 2). Twelve of these (Obs. 61–71, and 78) come from Skały, Site 1, Kielce District, being more than a half of all the samples from the locality of Skały (19). Sub-Class 1A also contains all observations from Góra Chełmowa, Kielce District (Obs. 2–3), the sole sample from Baszowice, Kielce District (Obs. 1), the sole observation from Pokrzywnica, Site 1, Starachowice District (Obs. 50), and two out of 10 observations from Rudki, Kielce District (Obs. 53–54). All these localities are situated less than 5 km from Skały. Other Holy Cross Mountains samples in this sub-class come from more distant localities (about 10 km), such as all three observations from Mychów, Site 1, Ostrowiec Świętokrzyski District (Obs. 32–34), four out of 15 observations from Łysa Góra, Kielce District (Obs. 17, 18, 27, and 29), two out of five observations from Jeleniów, Site 4, Kielce District (Obs. 5 and 9), and one out of six samples from Kunów, Site 1, Ostrowiec Świętokrzyski District (Obs. 15). There is also one observation from an unknown locality in the Holy Cross Mountains (Obs. 43).

**Fig 13:**
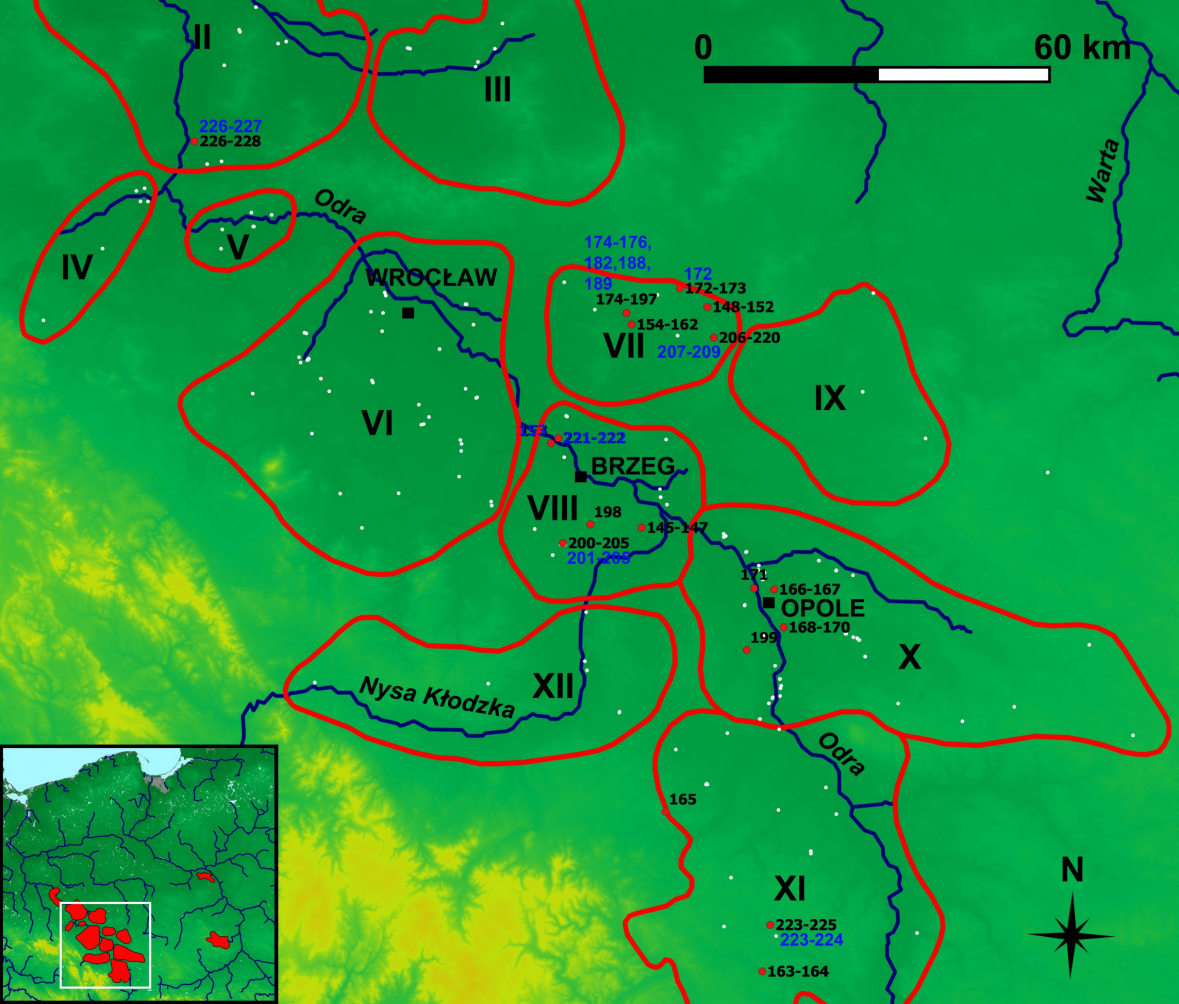
Geographical distribution of the Silesian samples from Sub-Class 1B (marked blue). Background map: *We acknowledge the use of imagery provided by services from NASA’s Global Imagery Browse Services (GIBS)*, *part of NASA’s Earth Observing System Data and Information System (EOSDIS)*. River network was re-drawn by G. Żabiński after FAO AQUASTAT, https://data.apps.fao.org/aquamaps/ Accessed 7 May 2023. This afterdrawing is similar but not identical to the original image and is therefore for illustrative purposes only.

Differences and similarities in the chemistry of samples from the same sites or localities can also be seen in the composition of the mostly Silesian sub-classes. In Sub-Class 2B (26 observations, with 21 Silesian), all the Silesian samples come from the Widawa region, but distances between individual localities may be even 20 km (Fig 4). The Silesian samples in this sub-class come from the following localities (all in the Namysłów District): Kowalowice (Obs. 148–149 –two out of five observations from this locality), Młokicie (Obs. 154–162 –all nine observations), Pawłowice (Obs. 173 –one out of two observations), Pielgrzymowice (Obs. 183, 184, 192–197 –eight out of 24 observations), and Rychnów (Obs. 219 –one out of 15 observations). Sub-Class 1B (28 observations, with 22 Silesian) gathers the finds from very distant localities (Fig 13), such as Sułków, Głubczyce District (Obs. 223–224 –two out of three observations), Pawłowice, Namysłów District (Obs. 172), Przylesie Dolne, Site 6, Brzeg District (Obs. 201–205 –five out of six observations), Tarchalice, Site 1, Wołów District (Obs. 226–227 –two out of three observations), Pielgrzymowice, Namysłów District (Obs. 174–176, 182, 188–189 –six out of 24 observations), Rychnów, Namysłów District (Obs. 207–209 –three out of 15 observations), and the sole observations from Lipki, Brzeg District (Obs. 153), Ryczyn Mały (Obs. 222) and Ryczyn Duży (Obs. 221) (both in the Oława District).

**Fig 14:**
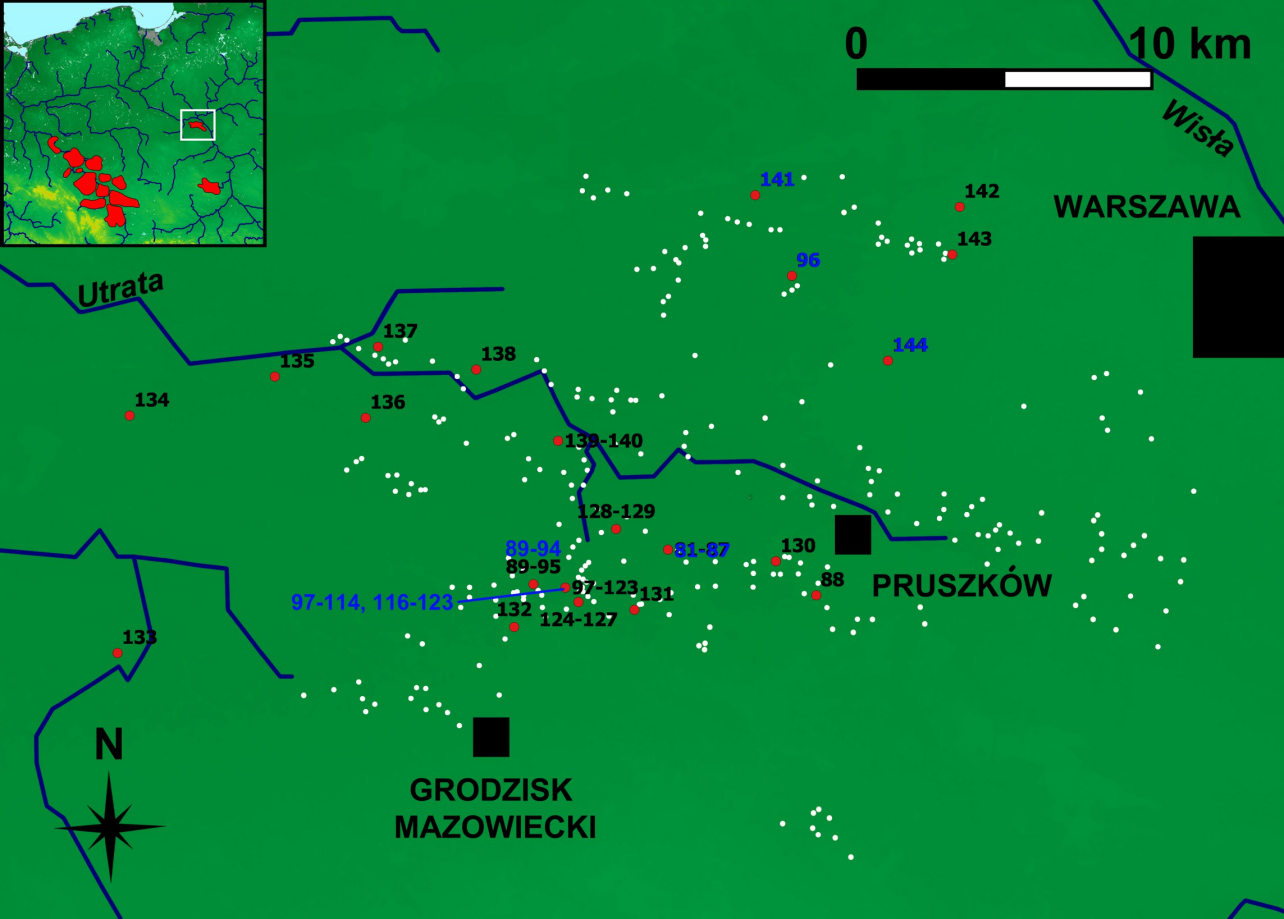
Geographical distribution of the Masovian samples from Sub-Class 7B (marked blue). Background map: *We acknowledge the use of imagery provided by services from NASA’s Global Imagery Browse Services (GIBS)*, *part of NASA’s Earth Observing System Data and Information System (EOSDIS)*. River network was re-drawn by G. Żabiński after FAO AQUASTAT, https://data.apps.fao.org/aquamaps/ Accessed 7 May 2023. This afterdrawing is similar but not identical to the original image and is therefore for illustrative purposes only.

In Sub-Class 7B (45 samples, with 42 Masovian), most Masovian samples come from Milanówek-Falęcin, Grodzisk Mazowiecki and Pruszków Districts (Obs. 97–114, 116–123–26 out of 31 samples). Seven samples are from Biskupice, Pruszków District (Obs. 81–87 –all from this locality), and six come from Kanie, Site 2, Pruszków District (Obs. 89–94 –out of seven samples). These localities (38 samples altogether) are within less than 5 km from Milanówek-Falęcin, and only three samples come from a more distant area (Kręczki, Zalesie-Wojcieszyn, and Ożarów Mazowiecki, all Warszawa-Zachód District, Obs. 96, 141, and 144 respectively) (Fig 14). Therefore, Sub-Class 7B is coherent both chemically and geographically.

### P levels in the iron smelting regions

Furthermore, regional differences in the P levels should be examined, as the P content was one of the main points in J. Piaskowski’s theory. P-low and P-high observations occur in all the regions, but differences between them are clear (Table 4). Most Holy Cross Mountains observations are P-low, both in the “clean” or “almost clean” sub-classes (98.31%), and in all the sub-classes together (88.75%). In contrast, the Masovian observations are mainly P-high (80.77 and 82.81% respectively). This result generally matches previous literature remarks that Masovian bog ores were in many cases P-rich [11, 17, 20, 79]. Most Silesian observations from the “clean” or “almost clean” classes are P-high, too (88.00%). In all the sub-classes, the number of P-high observations is still higher (61.90%), but a difference in the share of P-high and P-low observations is lower.

**Table 4:** Differences in P-levels according to P-low (A) and P-high (B) sub-classes in the observations from the three iron smelting regions of the Przeworsk Culture and from the Kraków-Częstochowa Jura.

**Observations in the “clean” or “almost clean” P-low (A) and P-high (B) sub-classes**			
Region	Number of observations	P-low (A)	P-high (B)
HCM	59	58 (98.31%)	1 (1.69%)
MAS	52	10 (19.23%)	42 (80.77%)
SIL	50	6 (12.00%)	44 (88.00%)
**Observations in all the P-low (A) and P-high (B) sub-classes**			
Region	Number of observations	P-low (A)	P-high (B)
HCM	80	71 (88.75%)	9 (11.25%)
MAS	64	11 (17.19%)	53 (82.81%)
SIL	84	32 (38.10%)	52 (61.90%)
JUR	12	10 (83.33%)	2 (16.67%)

HCM–Holy Cross Mountains; MAS–Masovia; SIL–Silesia; JUR–Jura.

### Possible addition of Ca-rich smelting fluxes

Contents of Ca in the samples must also be discussed. Some researchers argued that lime was intentionally added to the smelting batch in Masovia to dephosphorise metal, as most Masovian ores are P-high [17, 79]. Lime kilns in fact occur at Masovian smelting sites. They were used for roasting calcite-rich marlstone deposits to produce lime [17, 83] (see also Fig 5). Lime kilns also occur in Silesia. However, Sz. Orzechowski claimed that there was no evidence that these were anyhow related to iron smelting [11]. As demonstrated above, the PC graphs indicated a correlation between Ca and the Masovian observations (Fig 12). Moreover, considering the Ca raw values, it can be seen that although its maximum levels are higher in Silesia and in the Holy Cross Mountains, the median, the mean and the 3^rd^ quartile values for Masovia are evidently higher, both for all the observations together and solely for the slag observations (Table 5; see also S1 Dataset, Sheet “Boxplot Slag Ca RAW”).

**Table 5:** Ca levels (raw values) in all the observations and in the slag observations from the three iron smelting regions of the Przeworsk Culture and from the Kraków-Częstochowa Jura.

All observations	HCM	JUR	MAS	SIL
Nbr. of observations	80	12	64	84
Minimum	0.00	0.07	0.50	0.07
Maximum	6.05	2.93	4.61	13.36
1^st^ Quartile	0.21	0.56	1.57	0.93
**Median**	**0.79**	**0.95**	**2.12**	**1.36**
**3**^**rd**^ **Quartile**	**1.15**	**1.69**	**2.81**	**2.23**
**Mean**	**0.89**	**1.15**	**2.13**	**1.87**
Variance (n-1)	0.80	0.65	1.02	3.28
Standard deviation (n-1)	0.89	0.81	1.01	1.81
**Slag observations**	**HCM**	**JUR**	**MAS**	**SIL**
Number of observations	62	5	43	47
Minimum	0.00	0.48	1.00	0.16
Maximum	6.05	2.07	4.43	7.86
1^st^ Quartile	0.46	0.91	2.00	1.10
**Median**	**0.95**	**0.97**	**2.43**	**1.68**
**3**^**rd**^ **Quartile**	**1.26**	**1.72**	**3.00**	**2.35**
**Mean**	**1.03**	**1.23**	**2.56**	**2.03**
Variance (n-1)	0.89	0.42	0.51	1.81
Standard deviation (n-1)	0.94	0.65	0.71	1.35

HCM–Holy Cross Mountains; MAS–Masovia; SIL–Silesia; JUR–Jura.

Limestone was used in the blast furnace process and lime was also applied in the refining stage to moderate the presence of P [14, 36]. R. Pleiner observed that the CaO content in bloomery slag was usually c. 3–6% (which is similar to our data), while higher values (16–17%) were rare and could result from the contact of slag with Ca-rich furnace lining [14]. J. Piaskowski acknowledged the use of limestone in the blast furnace process to bind the excess of silica, but found any intentional lime addition in ancient Masovian bloomery smelting unlikely. In his opinion, the CaO content in slag was in fact higher in Masovia than in the Holy Cross Mountains (Table 5), but it was not higher than in many other slag finds from smelting regions where bog ores were used [20]. A possible use of CaCO_3_ to remove P was discussed by M. Thelemann and co-authors, who concluded that the presence of Ca oxides in slag could also be explained by the occurrence of Ca compounds in ores, furnace clay or ash [84]. Sz. Orzechowski proposed that the elevated Ca content in Masovian slag was related to the ore chemistry [11], while M. Woźniak has recently left this question open [83].

According to a recent research, the presence of lime in the bloomery furnace charge can theoretically contribute to a better yield, which is related to the replacement of FeO by CaO in the fayalite and wüstite, therefore leaving more iron oxide available for reduction. Nevertheless, a greater practical significance than achieving a highest possible yield can be attested in the production of a low-viscosity slag, as such slag favours the formation of a well-consolidated and easily forgeable bloom. In the FeO-SiO_2_-CaO system, this low-viscous slag is more likely to be wustite-rich [102].

In this regard, we focus solely on the raw values of Ca in the ores from the studied regions (Table 6; see also S1 Dataset, Sheets “Ores Ca RAW” and “Boxplot Ores Ca RAW”) to check whether they can in fact be responsible for the differences in the Ca levels in the slag observations. These data are of course not ideal, as most ore observations (and probably all for Masovia) refer to the ore samples collected from present-day geological outcrops.

**Table 6:** Ca levels in the ore samples from the three iron smelting regions of the Przeworsk Culture and from the Kraków-Częstochowa Jura.

Ore observations	HCM	JUR	MAS	SIL
Number of observations	18	7	21	37
Minimum	0.05	0.07	0.50	0.07
Maximum	2.07	2.93	4.61	13.36
1^st^ Quartile	0.17	0.53	0.57	0.71
**Median**	**0.20**	**0.94**	**0.86**	**1.14**
**3**^**rd**^ **Quartile**	**0.68**	**1.35**	**1.81**	**1.76**
**Mean**	**0.43**	**1.10**	**1.26**	**1.67**
Variance (n-1)	0.23	0.91	0.96	5.18
Standard deviation (n-1)	0.48	0.95	0.98	2.28

HCM–Holy Cross Mountains; MAS–Masovia; SIL–Silesia; JUR–Jura.

The Masovian ores are in fact Ca-richer than those from the Holy Cross Mountains or Jura, but do not significantly differ from the Silesian ores. Therefore, the ores were perhaps not solely responsible for the differences in Ca contents in the slag. It could be thus proposed that yet another Ca contribution may have come from Ca-rich Masovian clays used for the construction of furnaces. This may have been related to the local presence of marlstone deposits [17, 83]. On the other hand, the Ca levels in the Masovian slag do not indicate intentional limestone or lime addition to the smelting batch. Ca evidently plays a discriminant role for the Masovian observations, but further research is needed to explain its origin in the local slag.

## Conclusions

This study processed a “legacy database” of 240 analyses of slag and ore samples from the territory of the Przeworsk Culture using the contents of Al, Si, Ca, Mn, and P. The first aim was to evaluate the theory of the “Holy Cross Mountains metal” [18, 19]. We were only able to examine ore and slag, while it would be necessary to analyse iron artefacts, too, to fully assess the conclusions proposed by J. Piaskowski. However, we can offer a somewhat ambiguous response which says “yes and no.”

“Yes,” because the level of P is in fact an important discriminator between the Holy Cross Mountains and Masovia, as 88.75% of the Holy Cross Mountains observations are P-low, while 82.81% of the Masovian observations are P-high. Furthermore, over 66% of the observations from different regions were assigned to sub-classes which are dominated by the observations from one region only. Several such sub-classes were isolated for each region (Holy Cross Mountains–five; Masovia–two; Silesia–four). This result is archaeologically and geologically sound, as it cannot be expected that all ores and other components of smelting operations in one region will display similar chemistries. The overlap between the Holy Cross Mountains and Masovia (144 observations in total) is only 24 cases (16.67%), while 120 observations (83.33%) went to the sub-classes with no observations from the other region. The number of the Holy Cross Mountains observations in the “clean” or “almost clean” sub-classes is 73.75%. These findings indicate the relatively good discrimination between most of the Holy Cross Mountains samples and other regions, and are thus consistent with J. Piaskowski’s main conclusion. However, this theory must be further explored by future analyses of finds from Przeworsk Culture sites and from neighbouring regions.

Next, out of 193 multiple observations from the same sites or localities in all the regions, 164 (84.97%) went to the same sub-class. The match between slag and ore from the same sites (110 cases altogether) occurred in 28 cases (25.45%). As only a few variables were included in the analysis, the latter result is still not disappointing and confirms the usefulness of major elements in iron provenance studies. This result is perhaps also due to the fact that these major element contents are prone to some contamination from clay lining and charcoal, and due to the unlikelihood of the use of the same raw materials in all the smelting operations at a given site, especially at large and long-operating ones. The soundness of our results can also be seen at the level of individual sites or localities, where both similarities and differences between the chemistry of samples from such find places were confirmed.

However, the other part of the response is “no.” The level of P and observations based on metallographic analyses, which became crucial criteria of distinction in J. Piaskowski’s theory, are not sufficient to fully discriminate between all the main Przeworsk Culture ironmaking regions. The variable behaviour of P in the bloomery process and inconsistencies in the development of blacksmithing techniques [103] would not be sufficient for a reliable discrimination. The Holy Cross Mountains observations went to a few classes, so it is recommended to speak of the “Holy Cross Mountain metals,” rather than simply “metal.” Furthermore, an overlap still exists between P contents in the samples from Masovia and Silesia. It is therefore indispensable to also consider other elements to better discriminate between all these three regions.

Moreover, analyses that are based on major elements only would allow for the identification of the smelting systems rather than the absolute provenance to ores sources [36, 51, 104]. Therefore, due to the absence of the trace elemental analyses in our study and to the fact that nearly 2/3 of our observations were slag samples, our results mainly demonstrated the discrimination between metallurgical waste that was produced by regional smelting systems. Slag finds are particularly useful in provenance studies, as their chemistry can be less blurred than that of ores [50]. This, however, does not invalidate a postulate about the necessity of thorough prospection and sampling of ore deposits in order to assemble a collection that can be used as a reference signature.

As regards the other aim of our study, that is, developing the strategy for the new research with more complete data (major and trace elements), the main conclusion is a proper selection of the best discriminating variables, both for discrimination between metallurgical products as such and for provenance purposes. In this study, this issue was a simple one, as the number of variables was low. However, with major and trace elements together, the number of possible candidates will likely exceed 20. ANOVA proved to be a reliable tool, but with more variables it will be necessary to repeat this procedure several times (possibly not only for elements, but also for their ratios) to select a variable set with the best discriminant power. On the other hand, when the number of variables increases, more advanced feature selection methods might need to be considered [105]. This new research will hopefully produce a better separation of the Przeworsk Culture iron smelting regions. Ideally, a comprehensive study of this crucial problem of Roman Period East Germanic iron metallurgy should consider artefacts, blooms, smelting slag and all the components of iron smelting systems (ores, fuel, ash, clay, and possible fluxes), but this is a matter of a more distant future.

The present paper demonstrated that not only in studies focusing on the provenance of copper alloys (cf. [106, 107]), but also in the field of iron metallurgy, the so-called “legacy data” for slags and ores deserves consideration despite its limitations. The statistical groups distinguished here based on the processing of the large “legacy dataset” will lay a foundation for future studies that will pursue the objective of a refined identification of smelting signatures and ore provenance based on a larger number of elemental variables.

Another advantage of re-examining “legacy datasets” is the fact that some of the data produced in the past, when large-scale excavations of major ironmaking sites were possible, are often the only piece of information for those sites due to current unavailability of the physical samples. Although the new analytical methods can yield a higher number of trace elements, including potentially powerful ones (e.g., siderophile elements which are particularly enriched in iron ores and are characteristics of the geological conditions of their formation), or Os isotopic data, the fundamental significance of an access to physical samples is indisputable. For this reason, apart from developing new methods, it is recommended to re-examine results of previous analyses available for different parts of the world. Such re-examinations can bring interesting findings and suggest new paths for provenance studies. As remarked by P. Crew, “I have felt for a long time that both for slag analyses and geophysical surveys more information and better interpretations can be made, rather than the increasing tendency for more and more sophisticated and expensive techniques, which then can only be used by a fortunate few” (P. Crew, personal communication).

## Supporting information

S1 Data [53, 108].
(DOCX)

S1 Dataset Excel spreadsheet with the data and calculations in this study. (XLS)

S1 Text ANOVA textual detailed results. (TXT)

S1 Fig Density plots providing insight into the nature of the data used in this work. (TIF)

S1 File “Supplementary materials” manual; R codes (reproduction of all the ANOVA-based drawings included in this work; a user-friendly R-based toolbox for quickly generating a series of AHC trees; data imputation); csv data (analyses included in this study; dataset provided by G. Pagès and co-authors [53]; analyses related to data imputation; toy dataset demonstrating the required data format). (ZIP)
