# Correction: Old wine into new wineskins? “Legacy data” in research on Roman Period East Germanic iron smelting

**DOI:** 10.1371/journal.pone.0315700

**Published:** 2024-12-16

**Authors:** The PLOS ONE Staff

## Notice of Republication

This article was republished on October 25, 2023, to replace the S1 Dataset file, which had previously been a duplication of the S1 Data file. The publisher apologizes for the errors. Please download the supporting information files again to view the correct versions.

## References

[1] Żabiński G, Gramacki J, Gramacki A, Stepanov IS, Woźniak M (2023) Old wine into new wineskins? “Legacy data” in research on Roman Period East Germanic iron smelting. PLoS ONE 18(10): e0289771. 10.1371/journal.pone.0289771 37856549 PMC10586651

