# Supplementary material for: Old wine into new wineskins? “Legacy data” in research on Roman Period East Germanic iron smelting
Source: PLoS One. 2023 Oct 19;18(10):e0289771. doi: 10.1371/journal.pone.0289771 (PMC10586651; doi:10.1371/journal.pone.0289771)
Supplement: S1 Data — [53, 108]. (DOCX) [file pone.0289771.s001.docx]

All the data used in this paper, together with all calculations, are provided. All the ANOVA-based boxplots and density drawings can be easily reproduced using the attached R codes. The codes to easily perform the AHC or PCA-AHC analysis are a user friendly R-based solution (R toolbox) for quickly generating a series of dendrograms. These codes will allow to either separate production areas only, or to propose provenance hypotheses, if artefact data are available. This solution makes it possible to determine the origin of an unknown artefact based on the analysis of its occurrence in a series of successively built smaller dendrograms. In the present stage of development of the code, only one artefact observation can be analysed at one time. However, the analysis can easily be repeated separately for each observation for a given artefact. In order to analyse artefact data, the treatment is to be conducted in several steps. In the first step, a class with one observation for artefact data and production area data is selected. This class is used in the next step, and the procedure is repeated until a class solely containing the artefact observation and data on only one production area is obtained. If the artefact observation ends up in a class on its own or in a class with observations from more than one deposit, it can suggest that the source of metal for this artefact is not present in the dataset. A significant advantage of the proposed solution is its interactive nature. In each step, users can modify operating parameters to be applied in the next steps (for example, specifying the number of classes into which the data in the dendrogram should be divided). To facilitate the use of this method, it is recommended to pre-select the production areas and retain only those that are possible parent candidates on the basis of archaeological, historical, geographical and other grounds. This approach has been verified on the dataset provided by G. Pagès and co-authors [53] (for an approach similar to that discussed in our paper see also [108]). Additionally, codes for data imputation using the MICE PMM approach are provided.

All R codes and instructions on how to use them have been gathered as web-based reports prepared using the knitr R package (<https://yihui.org/knitr/>). This package is used worldwide for a dynamic report generation with R.
