# Supplementary figures and images for: Old wine into new wineskins? “Legacy data” in research on Roman Period East Germanic iron smelting
Source: PLoS One. 2023 Oct 19;18(10):e0289771. doi: 10.1371/journal.pone.0289771 (PMC10586651; doi:10.1371/journal.pone.0289771)

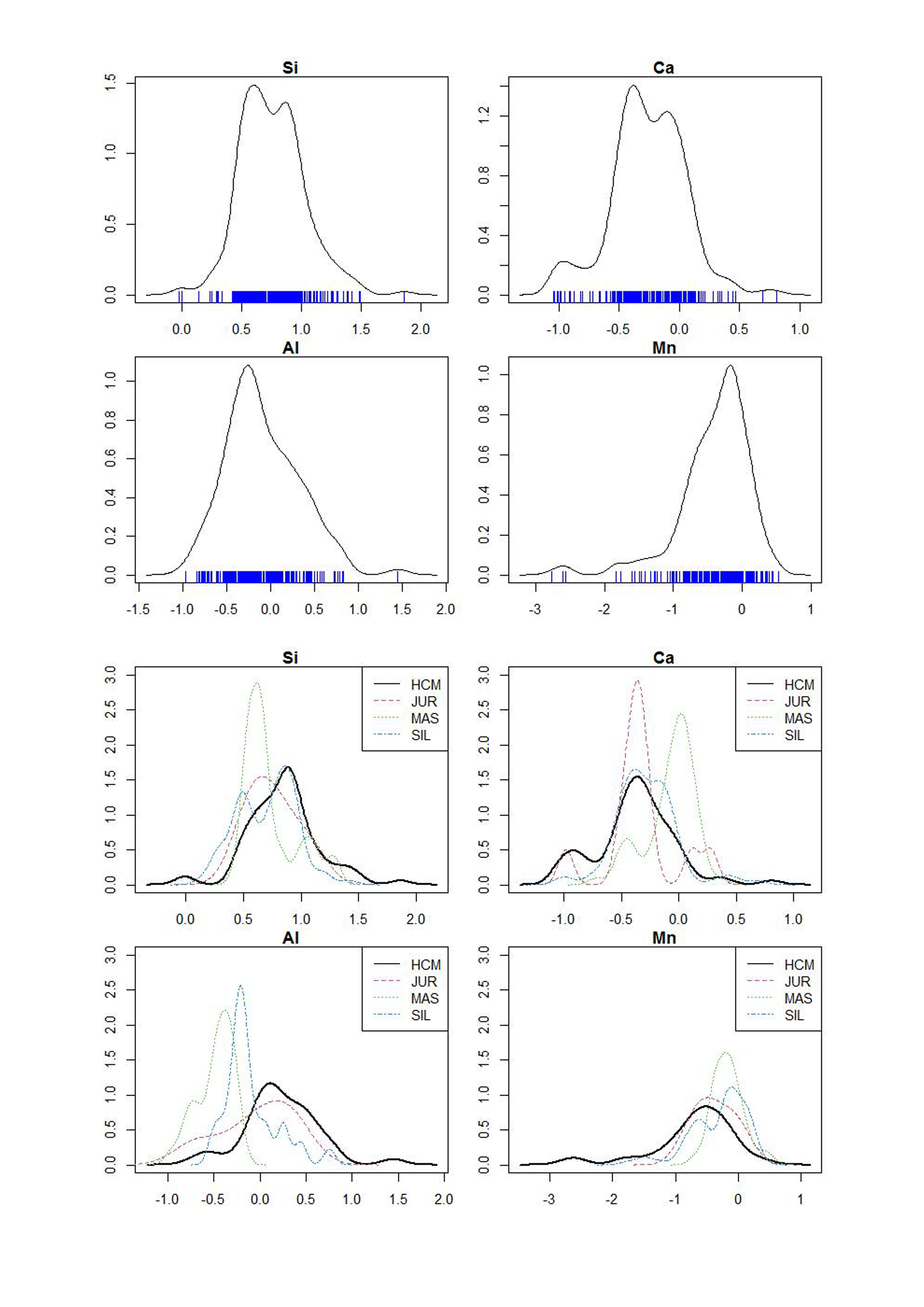

Supplement: S1 Fig — (TIF) [file pone.0289771.s004.tif]

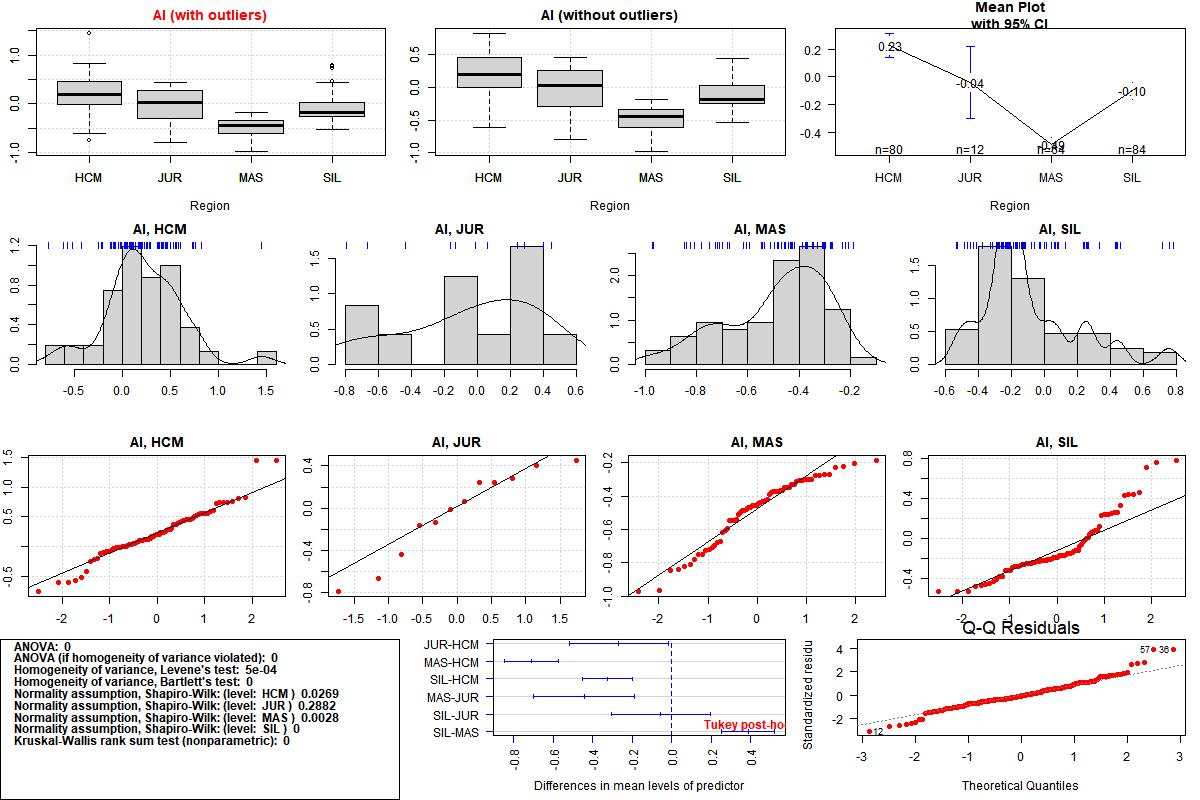

Supplement: S1 File — (ZIP) [file pone.0289771.s005.zip › S4 R Materials/figs/Al_ANOVA.jpeg]

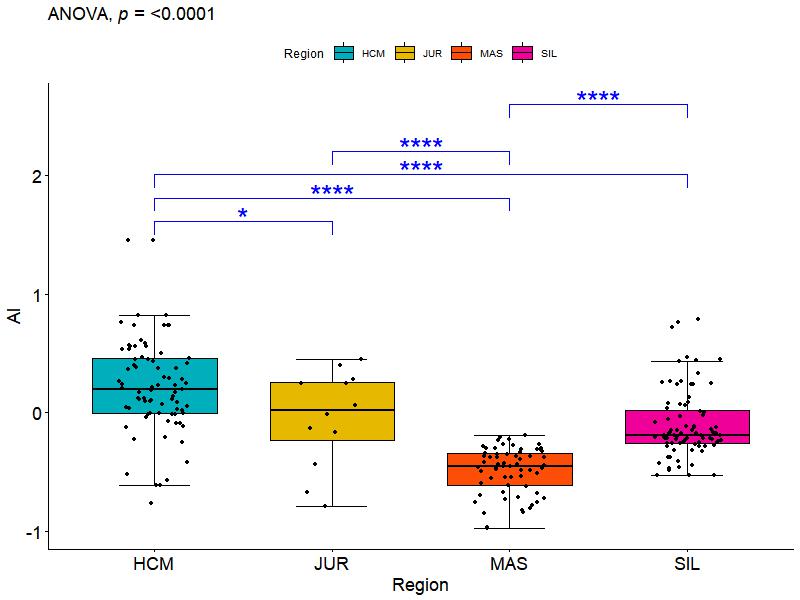

Supplement: S1 File — (ZIP) [file pone.0289771.s005.zip › S4 R Materials/figs/Al_boxplots.jpeg]

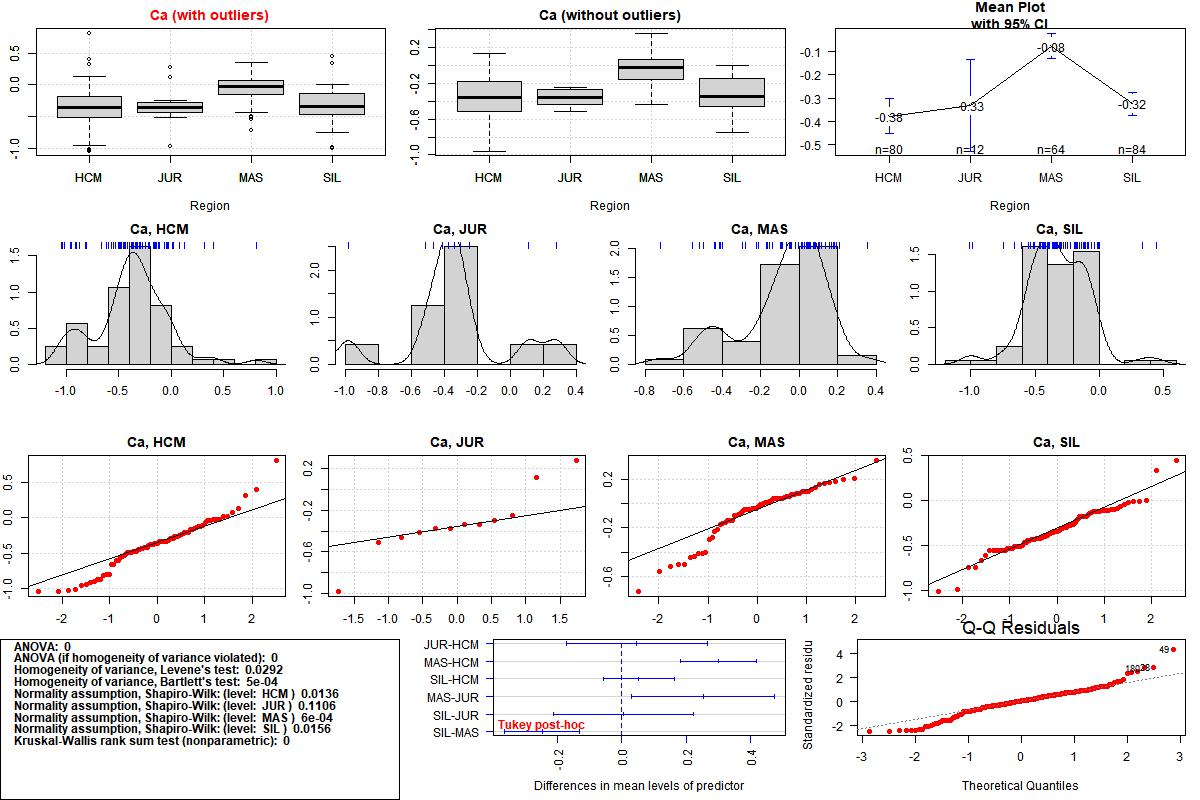

Supplement: S1 File — (ZIP) [file pone.0289771.s005.zip › S4 R Materials/figs/Ca_ANOVA.jpeg]

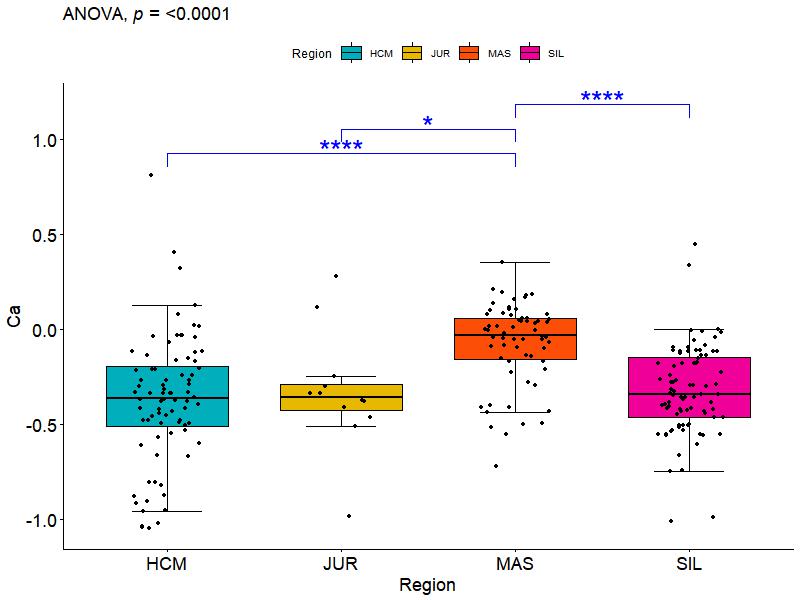

Supplement: S1 File — (ZIP) [file pone.0289771.s005.zip › S4 R Materials/figs/Ca_boxplots.jpeg]

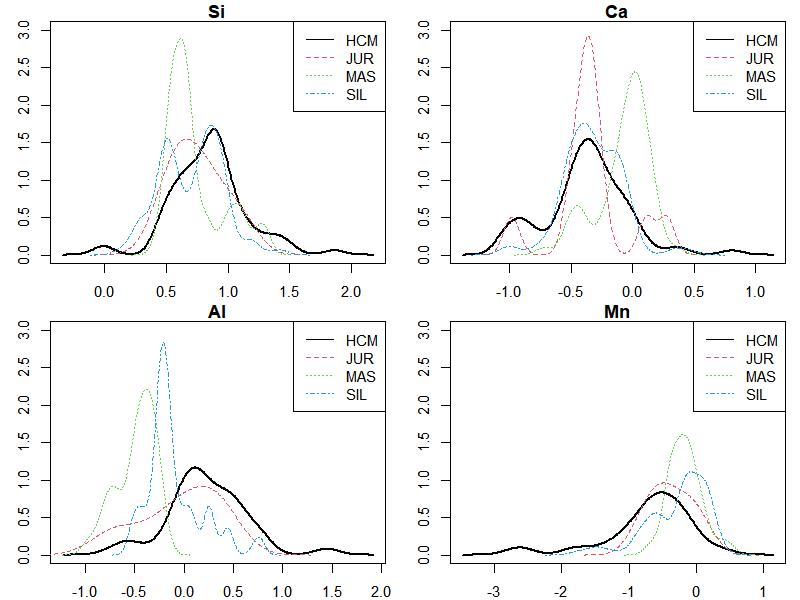

Supplement: S1 File — (ZIP) [file pone.0289771.s005.zip › S4 R Materials/figs/density_by_region.jpeg]

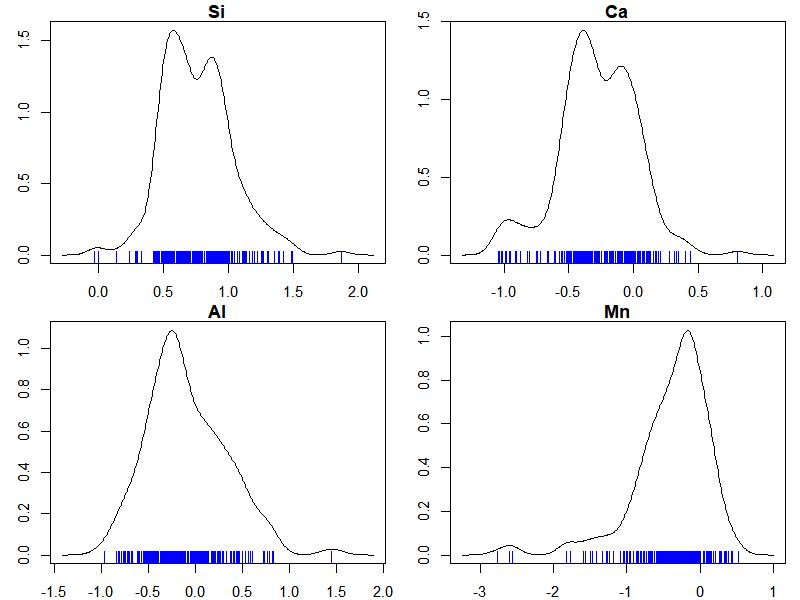

Supplement: S1 File — (ZIP) [file pone.0289771.s005.zip › S4 R Materials/figs/density_total.jpeg]

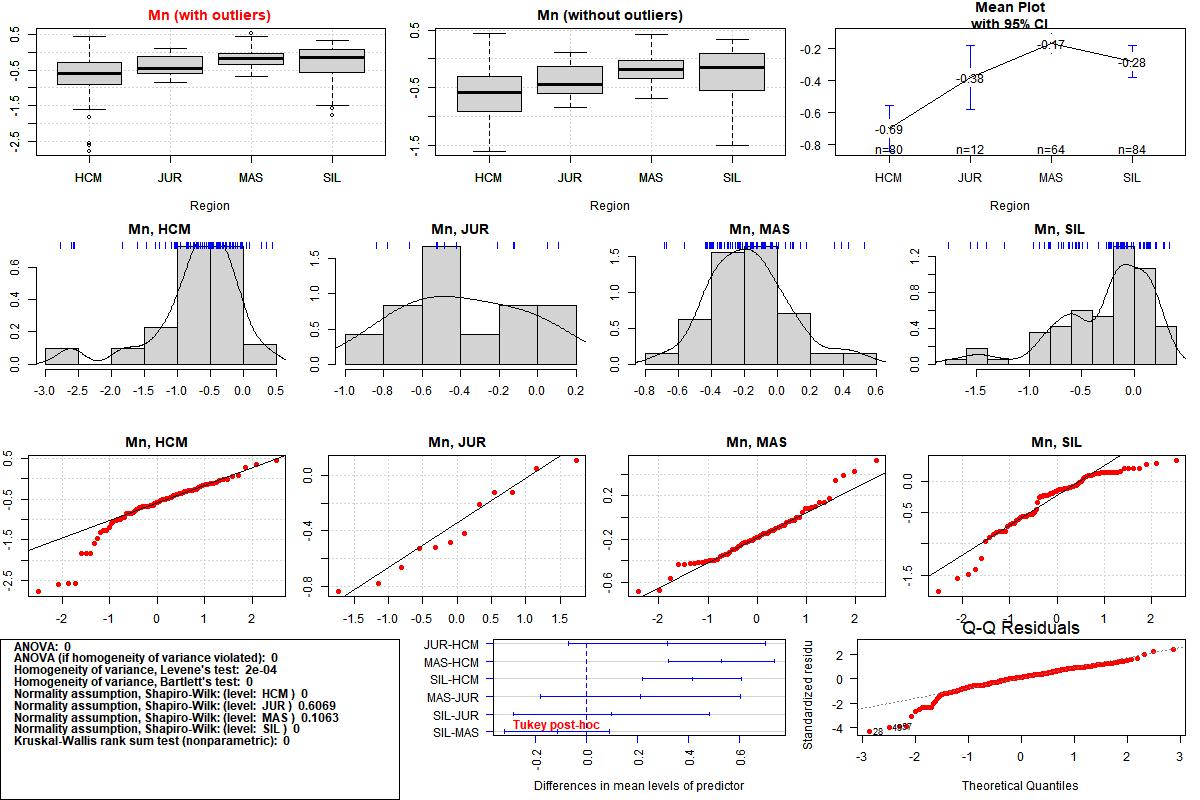

Supplement: S1 File — (ZIP) [file pone.0289771.s005.zip › S4 R Materials/figs/Mn_ANOVA.jpeg]

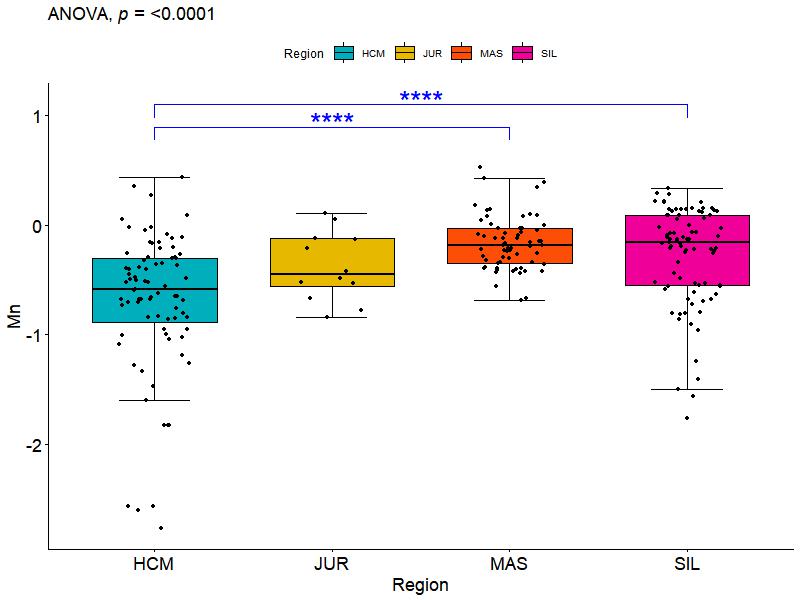

Supplement: S1 File — (ZIP) [file pone.0289771.s005.zip › S4 R Materials/figs/Mn_boxplots.jpeg]

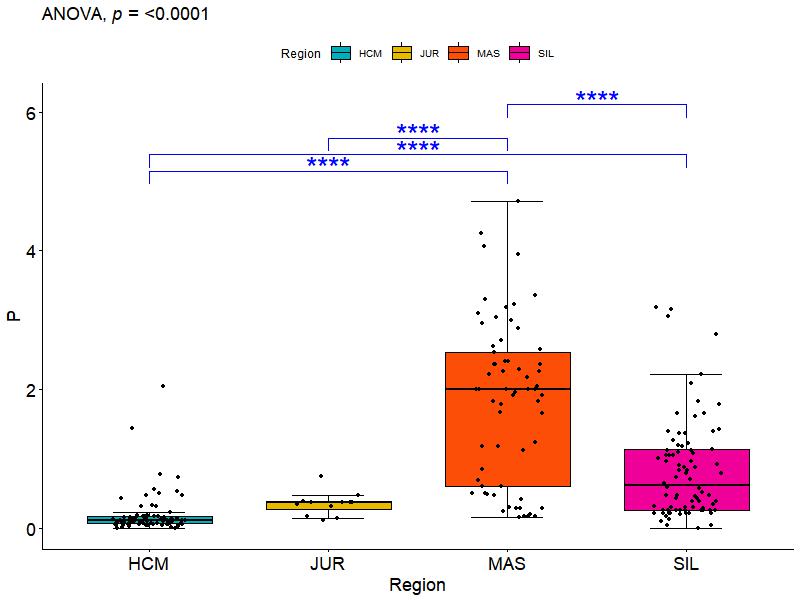

Supplement: S1 File — (ZIP) [file pone.0289771.s005.zip › S4 R Materials/figs/P_boxplots.jpeg]

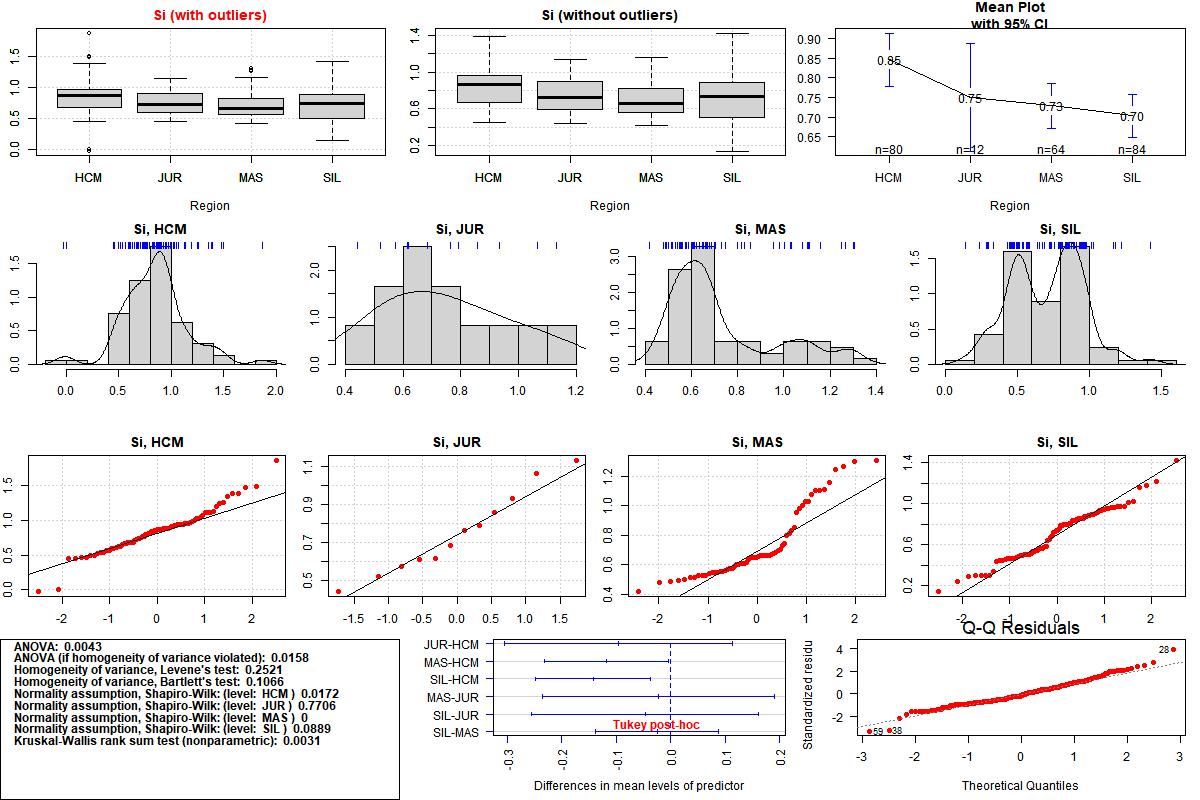

Supplement: S1 File — (ZIP) [file pone.0289771.s005.zip › S4 R Materials/figs/Si_ANOVA.jpeg]

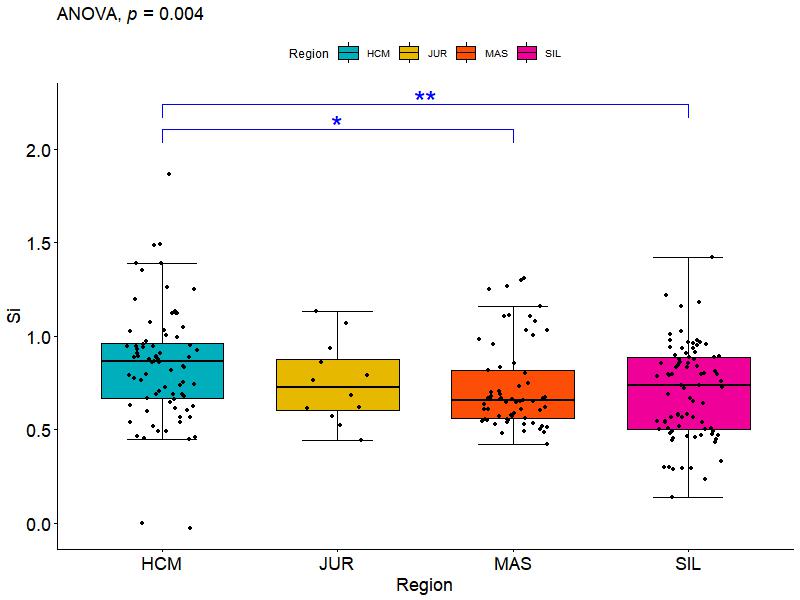

Supplement: S1 File — (ZIP) [file pone.0289771.s005.zip › S4 R Materials/figs/Si_boxplots.jpeg]

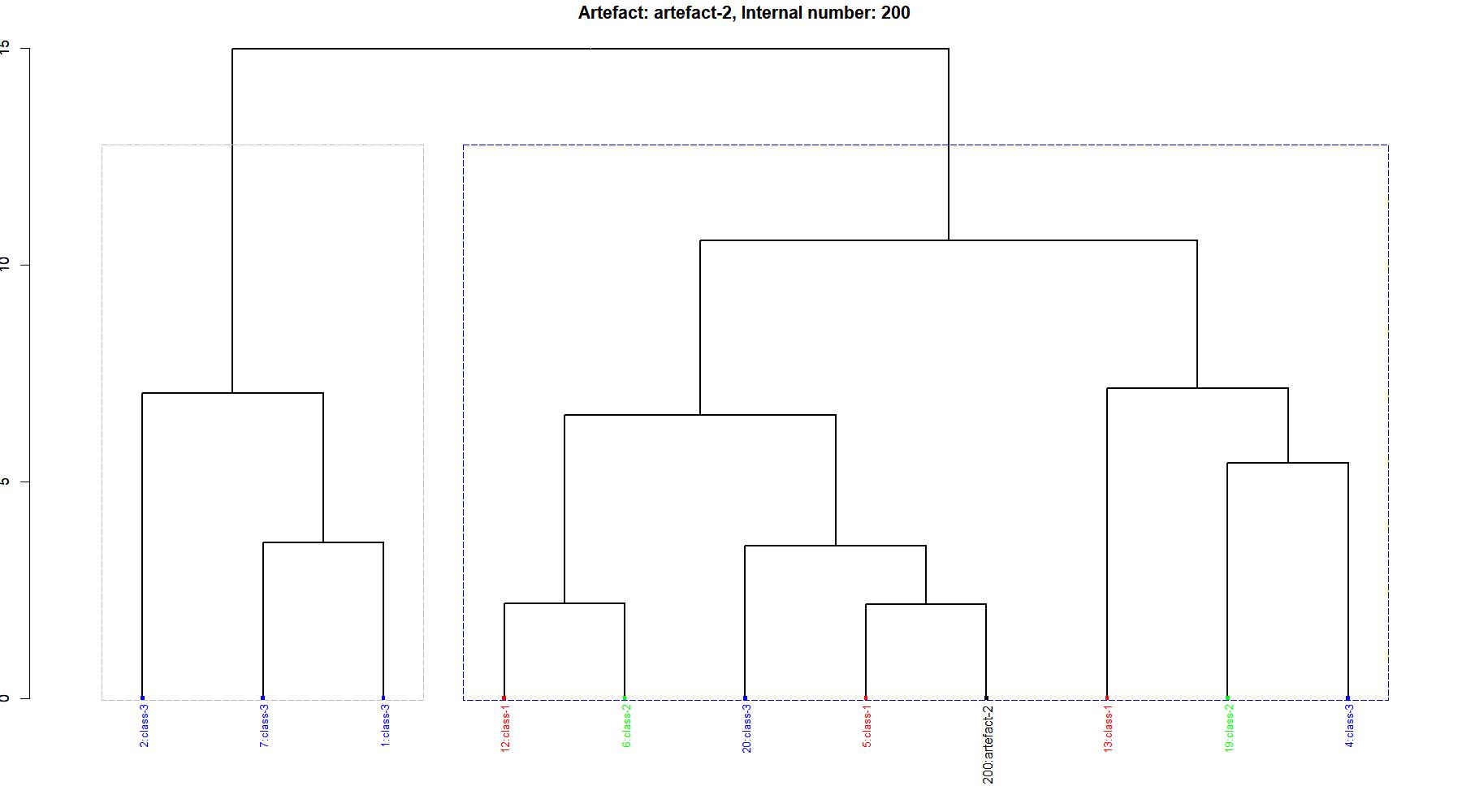

Supplement: S1 File — (ZIP) [file pone.0289771.s005.zip › S4 R Materials/figs_sifter/artefact_obs_No_200_fig_1.jpeg]

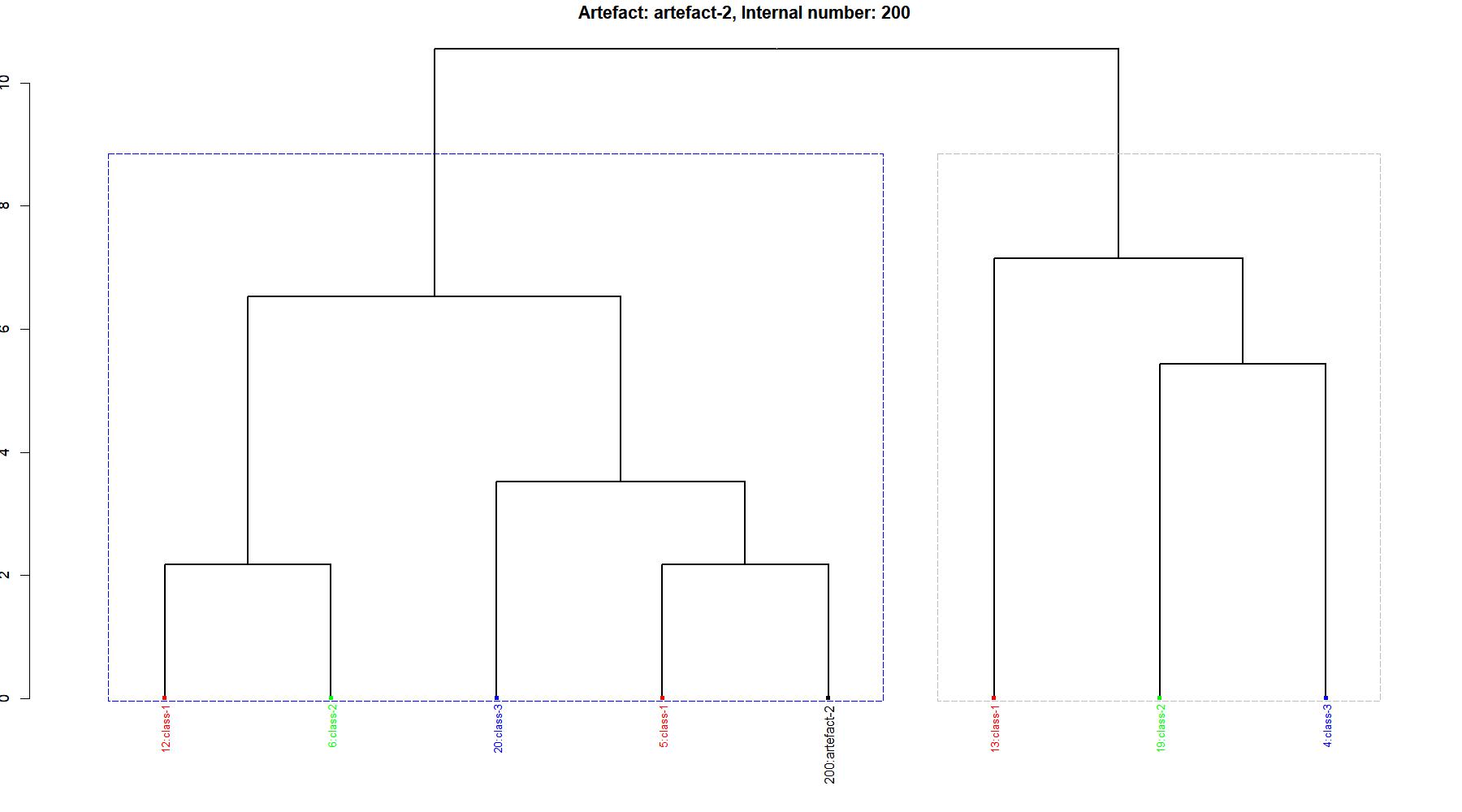

Supplement: S1 File — (ZIP) [file pone.0289771.s005.zip › S4 R Materials/figs_sifter/artefact_obs_No_200_fig_2.jpeg]

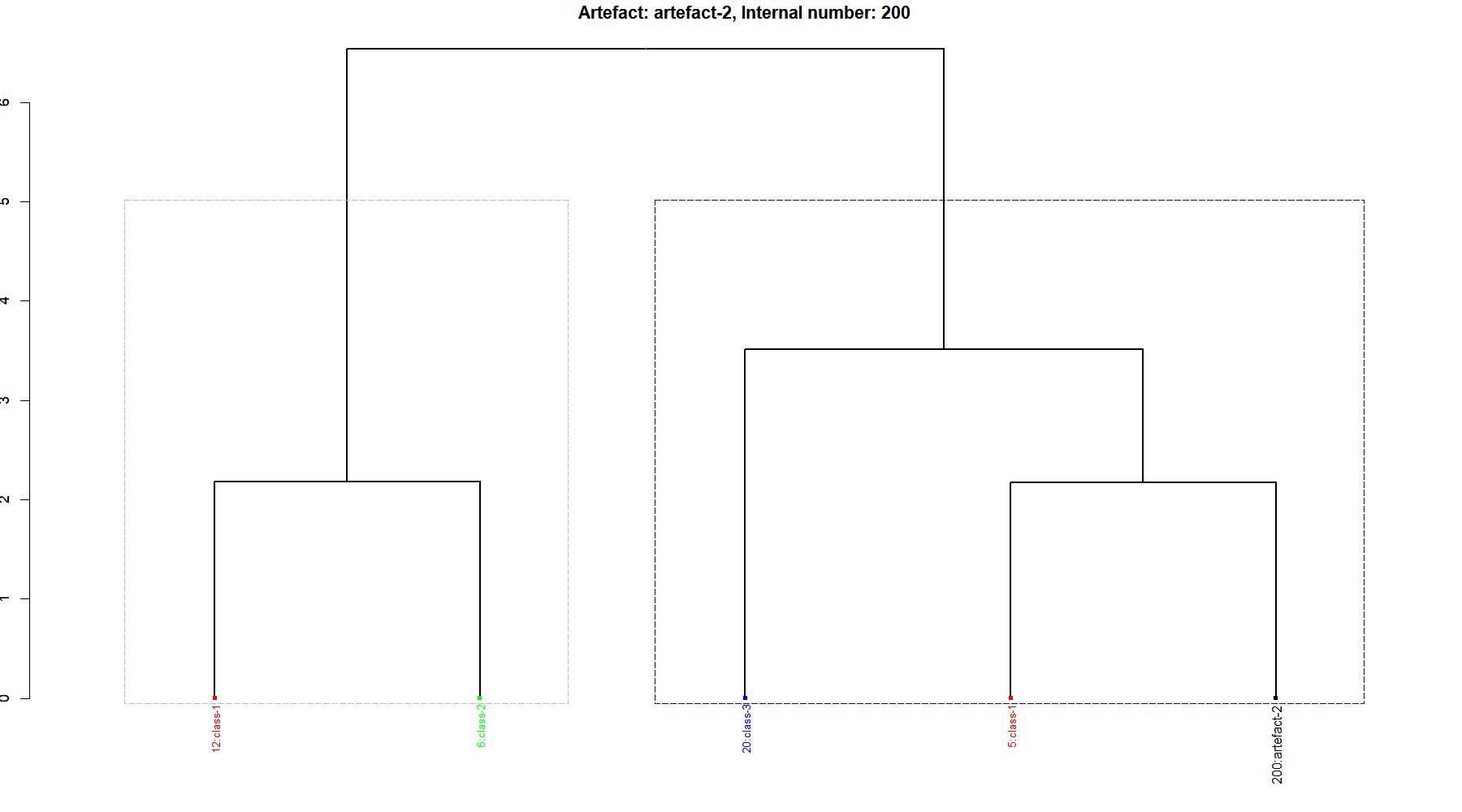

Supplement: S1 File — (ZIP) [file pone.0289771.s005.zip › S4 R Materials/figs_sifter/artefact_obs_No_200_fig_3.jpeg]

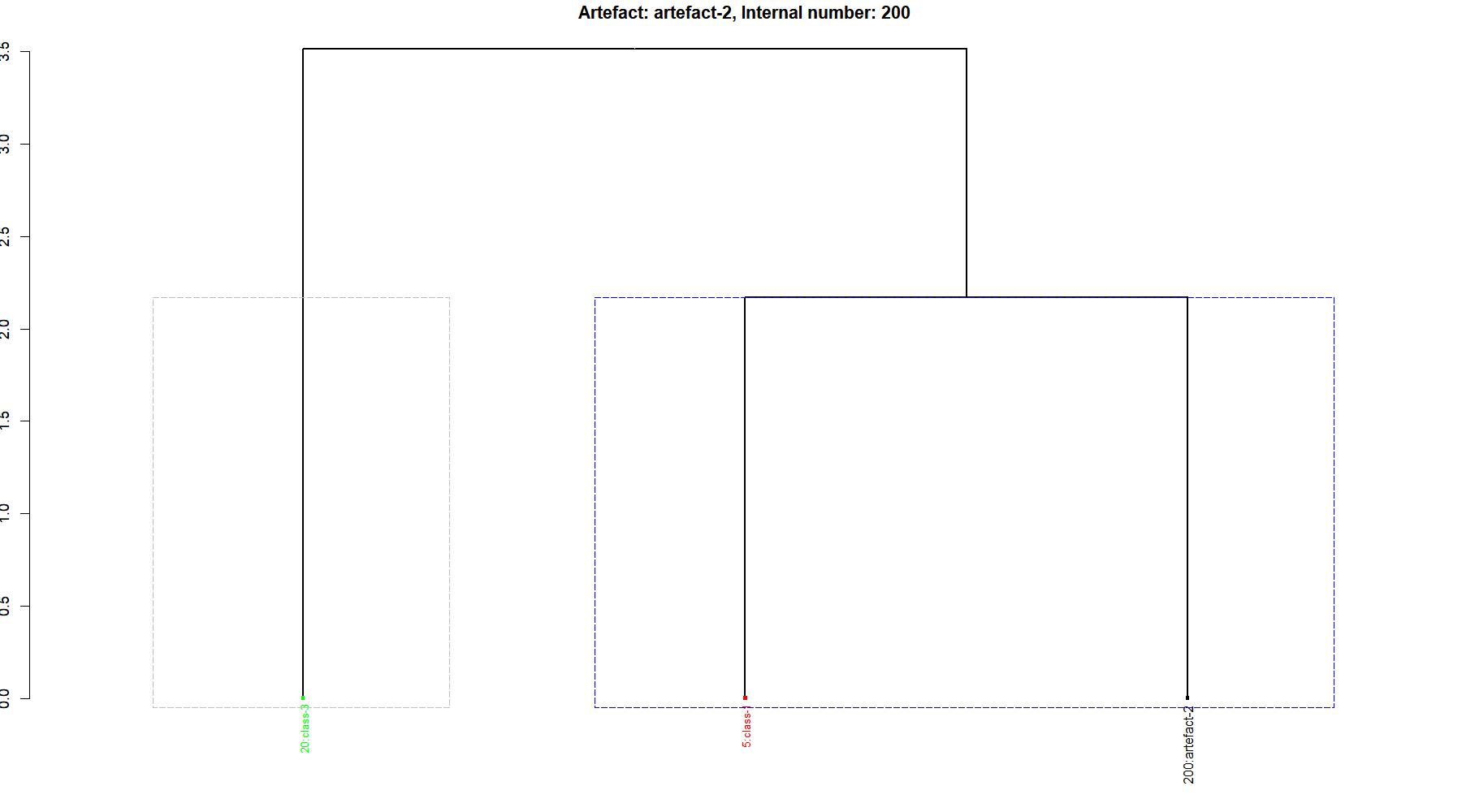

Supplement: S1 File — (ZIP) [file pone.0289771.s005.zip › S4 R Materials/figs_sifter/artefact_obs_No_200_fig_4.jpeg]

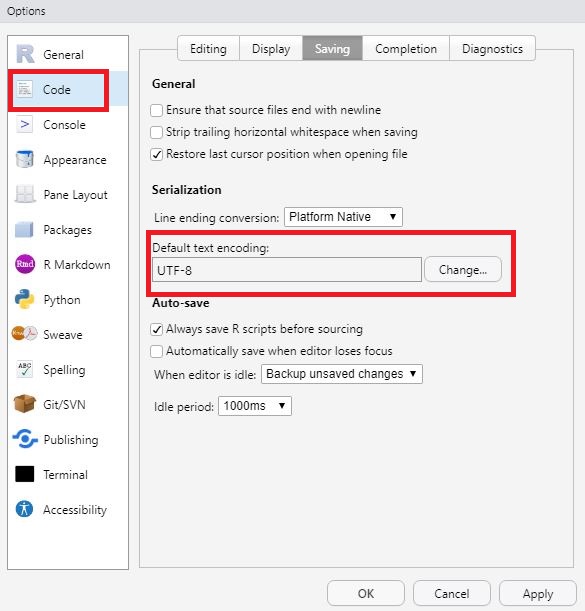

Supplement: S1 File — (ZIP) [file pone.0289771.s005.zip › S4 R Materials/screenshot/RStudio_screenshot.jpeg]
