# Supplementary material for: Old wine into new wineskins? “Legacy data” in research on Roman Period East Germanic iron smelting
Source: PLoS One. 2023 Oct 19;18(10):e0289771. doi: 10.1371/journal.pone.0289771 (PMC10586651; doi:10.1371/journal.pone.0289771)
Supplement: S1 File — (ZIP) [file pone.0289771.s005.zip › S4 R Materials/Supplementary_Materials.html]

Supplementary materials in conjunction with the paper Old Wine into New Wineskins? “Legacy Data” in Research on East Germanic Iron Smelting


# Supplementary materials in conjunction with the paper **Old Wine into New Wineskins? “Legacy Data” in Research on East Germanic Iron Smelting**

# 1 R version used and time of report generation

```
Generated on: 17-05-2023
R version:    4.3.0
```

# 2 Preliminary remarks, required packages, scripts and functions.

Back to top

All the R codes have been tested in R version **4.3.0**. Most likely, however, they can also be launched in other versions of R (probably not older than **4.x**, the codes were not tested on very old **3.x** releases).

We strongly recommend using **RStudio** program to work with R (https://www.rstudio.com). For trouble-free reading of data files containing national characters, we suggest that you set the default character encoding in RStudio to UTF-8 (menu `Tools -> Global options -> Code -> Saving`), see the below screenshot.

Figure 2.1: A screenshot from RStudio showing how to set the default character encoding to UTF-8

Before running our codes, a couple of required packages **must be installed manually** and then **loaded** using the following commands:

```
# Install and load required R packages
pkgs <- c("ks", "car", "MASS", "gplots", "dunn.test", "DescTools", 
          "dendextend", "RColorBrewer", "ggpubr", "dplyr", "rstatix",
          "mice", "grDevices", "ggplot2")

to_install = !pkgs %in% installed.packages()

if(any(to_install)) {
  install.packages(pkgs[to_install])
}

library(ks) 
library(car)
library(MASS)
library(gplots)
library(dunn.test)
library(DescTools)
library(dendextend)
library(RColorBrewer)
library(ggpubr)
library(dplyr)
library(rstatix)
library(mice)
library(grDevices)
library(ggplot2)
```

In the last step, we need to load a script with some dedicated functions

```
source("funs.R")
```

# 3 Replication of figures

Back to top

## 3.1 Read the required dataset

```
source("read_data_for_ANOVA.R")
head(przeworsk_clr)
```

```
  ObsNo       Period           FindType OreType FindType2                  Site Region             RegionSite        Si         Ca          Al      K     Mg         Mn      P PFiltr
1     1 Roman Period               Slag    <NA>         S             Baszowice    HCM        Baszowice_S_HCM 0.6679523 -0.4557615  0.17593752 0.0415 0.5487 -0.3881283 0.1484      ?
2     2 Roman Period               Slag    <NA>         S         Chełmowa Góra    HCM    Góra Chełmowa_S_HCM 0.6892065 -0.3805857 -0.01270813 0.0415 0.5186 -0.2959126 0.0970      ?
3     3 Roman Period               Slag    <NA>         S         Chełmowa Góra    HCM    Góra Chełmowa_S_HCM 0.6892065 -0.3805857 -0.01270813 0.0415 0.5186 -0.2959126 0.0786      ?
4     4 Roman Period               Slag    <NA>         S Góra Jeleniowska pass    HCM Góra Jeleniowska_S_HCM 0.4475520 -0.3590326  0.02289661 0.0415 1.1819 -0.1114160 0.1500      ?
5     5 Roman Period Ore. hematite dust    <NA>         O      Jeleniów. Site 4    HCM         Jeleniów_O_HCM 0.6275739 -0.3944675  0.25010535 0.0415 0.1025 -0.4832117 0.0655      ?
6     6 Roman Period               Slag    <NA>         S      Jeleniów. Site 4    HCM         Jeleniów_S_HCM 1.0235435 -0.2186419  0.20269720 0.0415 0.2231 -1.0075987 0.0864      ?
```

## 3.2 Replication of Figures 6, 7, 8 and 9

Back to top

Run the following script:

```
source("figs_ANOVA.R")
```

After executing this script in the `figs` directory four `jpeg` files will be created.

```
Al_ANOVA.jpeg 
Ca_ANOVA.jpeg 
Mn_ANOVA.jpeg 
Si_ANOVA.jpeg
```

Below we display one selected `jpeg` file (for `Al` values). The other three are analogous.

Figure 3.1: ANOVA results for Al values in the three iron smelting regions of the Przeworsk Culture and in the Kraków-Częstochowa Jura. HCM – Holy Cross Mountains; MAS – Masovia; SIL – Silesia; JUR – Jura

## 3.3 Replication of Figures 10 and 11

Back to top

Run the following script:

```
source("figs_boxplots.R")
```

After executing this script in the `figs` directory five `jpeg` files will be created.

```
Al_boxplots.jpeg 
Ca_boxplots.jpeg 
Mn_boxplots.jpeg 
P_boxplots.jpeg 
Si_boxplots.jpeg
```

Below we display one selected `jpeg` file (for `Al` values). The other five are analogous.

Figure 3.2: Detailed boxplots with Al values in the three iron smelting regions of the Przeworsk Culture and in the Kraków-Częstochowa Jura. HCM – Holy Cross Mountains; MAS – Masovia; SIL – Silesia; JUR – Jura. Blue asterisks indicate regional differences in the levels of each element

# 4 Density plots

Back to top

The figures presented in section Replication of figures are supplemented with data density plots, which provide additional information about the nature of the analysed data.

```
source("figs_density.R")
```

Figure 4.1: Total densities in every variable (Si, Ca, Al, Mn)

Figure 4.2: Comparision of densities in regions. HCM – Holy Cross Mountains; MAS – Masovia; SIL – Silesia; JUR – Jura

# 5 A user friendly R-based toolbox for quickly generating a series of AHC trees

Back to top

We will demonstrate the operation of this toolbox on an artificial and very small dataset. This will make it easier for the reader to understand how it works. Then we will run analogous examples using the target dataset.

## 5.1 Data format

Back to top

This section explains the required data format and can be helpful when you want to prepare your own dataset for your own analysis. The following rules must be observed:

- the input data must be a data frame,
- the first `n` columns contain the data to be analysed,
- the `n + 1` column contains the class identifier,
- the `n + 2` column contains the unique ID of individual records,
- the rest of columns is not analysed and can contain any data (but our sample data does not contain any additional data),
- the subsequent columns must be separated by tab characters.

The demonstration is based on two toy datasets where `n = 4`. The first contains training data, the second contains artefact observations to be analysed. To begin with, let us load two datasets into R.

```
toy <- read.csv(file = "data/toy_example.csv", dec = ".", sep = "\t", header = TRUE)
toy_artefact <- read.csv(file = "data/toy_example_artefacts.csv", dec = ".", sep = "\t", header = TRUE)
```

```
    A    B    C    D   Class UniqueID
 3.18 9.70 8.30 5.10 class-1       12
 8.39 9.69 7.37 8.67 class-1       13
 1.79 8.45 4.40 6.58 class-1        5
 1.17 9.55 7.53 5.39 class-2        6
 7.43 9.50 2.22 6.02 class-2       19
 3.13 1.36 6.67 5.33 class-3        7
 6.25 7.15 5.13 2.27 class-3        4
 2.29 6.31 4.69 7.05 class-3       20
 6.37 1.89 7.74 4.38 class-3        1
 8.67 2.46 2.61 4.78 class-3        2
```

```
    A    B    C    D      Class UniqueID
 2.18 8.71 3.30 4.20 artefact-1      100
 1.39 9.22 3.37 4.88 artefact-2      200
```

The columns in both datasets must have the same names (we will check this below). In addition, the column where the class names are must be defined as factor (we will set it right now).

```
colnames(toy) == colnames(toy_artefact)
```

```
[1] TRUE TRUE TRUE TRUE TRUE TRUE
```

```
toy$Class <- as.factor(toy$Class) 
toy_artefact$Class <- as.factor(toy_artefact$Class)

str(toy$Class)
```

```
 Factor w/ 3 levels "class-1","class-2",..: 1 1 1 2 2 3 3 3 3 3
```

```
str(toy_artefact$Class)
```

```
 Factor w/ 2 levels "artefact-1","artefact-2": 1 2
```

## 5.2 Demonstration

Back to top

After loading all required functions we can start demonstrating the capabilities of the toolbox. To begin with, we call the function in its simplest form. We will get a tree as a result. Elements from each class are in a different colour. The `mai_bottom` parameter controls the amount of free space at the bottom of the drawing. When class names are long, it is worth setting a larger value (you need to test it practically).

```
source("funs.R")

out <- sifter(
  data_main = toy, 
  class_col = 5, 
  internal_number_col = 6,
  mai_bottom = 0.8,
  verbose = TRUE)
```

Elements belonging to the selected class may be displayed in slightly larger black font. The highlighted class name is displayed as the title of the plot.

```
out <- sifter(
  data_main = toy, 
  class_col = 5, 
  internal_number_col = 6,
  class_to_highlight = "class-2",
  mai_bottom = 0.8,
  verbose = TRUE)
```

The next demonstration draws rectangles around the branches of a dendrogram highlighting the corresponding clusters. First the dendrogram is cut at a certain level (according to the input parameter `num_of_classes`), then rectangles are drawn around selected branches.

```
out <- sifter(
  data_main = toy, 
  class_col = 5, 
  internal_number_col = 6,
  num_of_classes = 3,
  mai_bottom = 0.8,
  verbose = TRUE)
```

In this example the dataset with artefact observations is used. The effect of calling the function is similar to the previous one. This time, however, the class where the artefact observation is located is surrounded by a blue rectangle. The artefact observation name is displayed in slightly larger black font.

```
out <- sifter(
  data_main = toy, 
  data_art = toy_artefact[2,],
  class_col = 5, 
  num_of_classes = 3,
  internal_number_col = 6,
  mai_bottom = 0.9,
  verbose = TRUE)
```

In the last example the dendrogram is cut at a certain **height** of the tree (parameter `cutting_at_height` is used here). A red horizontal line is drawn at the specified value (9 in this example) and the relevant number of rectangles is drawn (3 in this example).

```
out <- sifter(
  data_main = toy, 
  class_col = 5, 
  internal_number_col = 6,
  cutting_at_height = 9,
  mai_bottom = 0.8,
  verbose = TRUE)
```

## 5.3 Running `sifter()` function in a loop

Back to top

The `sifter()` function can either separate production areas only (as demonstrated above), or to propose provenance hypotheses, if artefact data is available. This solution makes it possible to determine the origin of an unknown artefact based on the analysis of its occurrence in a series of successively built smaller dendrograms. In order to analyse artefact data, the treatment is to be conducted in several steps. In the first step, a class with one observation for artefact data and observations for production area data is selected. This class is used in the next step, and the procedure is repeated until a class solely containing the artefact observation and data on only one production area is obtained.

```
par(mfrow = c(2,2), pty = "m")
par(mai = c(0.1, 0, 0, 0))

# -------
# Step 1
#--------
# The first dendrogram is generated. The class where the artefact 
# observation is located is surrounded by a blue rectangle
out <- sifter(
  data_main = toy, 
  data_art = toy_artefact[2,],
  class_col = 5, 
  num_of_classes = 2,
  internal_number_col = 6,
  mai_bottom = 0.8,
  verbose = TRUE) 

# -------
# Step 2
# -------
# Now, the data from the blue rectangle from Step 1 become inputs 
# to the sifter() function. And again, The class where the artefact 
# observation is located is surrounded by a blue rectangle.
# The artefact Observation is still in a heterogeneous class, so another 
# step is required,
r <- which(rownames(out$data_with_artefact) == out$artefact_row)
toy_2 <- out$data_with_artefact[-r, ]
toy_artefact_2 <- out$data_with_artefact[r, ]
out <- sifter(
  data_main = toy_2, 
  data_art = toy_artefact_2, 
  class_col = 5, 
  internal_number_col = 6,
  num_of_classes = 2,
  mai_bottom = 0.8,
  verbose = TRUE) 

# -------
# Step 3
# -------
# After completing the next step, the artefact observation is still in a 
# heterogeneous class, so another step is required.
r <- which(rownames(out$data_with_artefact) == out$artefact_row)
toy_2 <- out$data_with_artefact[-r, ]
toy_artefact_2 <- out$data_with_artefact[r, ]
out <- sifter(
  data_main = toy_2, 
  data_art = toy_artefact_2, 
  class_col = 5, 
  internal_number_col = 6,
  num_of_classes = 2,
  mai_bottom = 0.8,
  verbose = TRUE) 

# -------
# Step 4
# -------
# The fourth step ends up in a homogenous class which ends the analysis.
r <- which(rownames(out$data_with_artefact) == out$artefact_row)
toy_2 <- out$data_with_artefact[-r, ]
toy_artefact_2 <- out$data_with_artefact[r, ]
out <- sifter(
  data_main = toy_2, 
  data_art = toy_artefact_2, 
  class_col = 5, 
  internal_number_col = 6,
  num_of_classes = 2,
  mai_bottom = 0.8,
  verbose = TRUE)
```

```
out$is_homogenous
```

All the above steps can be enclosed inside an another function `sifter_toolbox()` and the 4 above steps can now be run subsequently. As a result, four `jpeg` files are created in the `figs_sifter` directory. In addition, a text file is created where detailed results from every step are saved.

```
sifter_toolbox(
  data_main = toy, 
  data_art = toy_artefact[2,],
  class_col = 5,
  internal_number_col = 6,
  num_of_classes = 2
)
```

```
artefact_obs_No_200_fig_1.jpeg 
artefact_obs_No_200_fig_2.jpeg 
artefact_obs_No_200_fig_3.jpeg 
artefact_obs_No_200_fig_4.jpeg 
artefact_obs_No_200_results_step_by_step.txt
```

```
          step_1         step_2         step_3         step_4
1     12:class-1     12:class-1     20:class-3      5:class-1
2      6:class-2      6:class-2      5:class-1 200:artefact-2
3     20:class-3     20:class-3 200:artefact-2               
4      5:class-1      5:class-1                              
5 200:artefact-2 200:artefact-2                              
6     13:class-1                                             
7     19:class-2                                             
8      4:class-3
```

## 5.4 Experiments using the dataset provided by G. Pagès and co-authors [53]

Back to top

First load the required datasets (main and with the artefacts). Everything else is analogous as shown in the previous section.

```
source("read_data_PAGES.R")
head(train_p)
```

```
         Ce         Eu         Hf        La         Nb        Nd           Pr          Sm          U         Y         Yb         Cs  Region ObsExcel
1 0.9455778 -0.2686046 -0.7088956 0.6232596 -0.2954862 0.5797839  0.002399023 -0.05685725 -0.3528519 0.4939541 -0.3160180 -0.6462609 Canigou        1
2 0.9489712 -0.2155470 -0.7742139 0.6461330 -0.4009617 0.6191837  0.038274381 -0.02468314 -0.3063963 0.5462085 -0.2488320 -0.8281367 Canigou        2
3 0.9188176 -0.3948016 -0.6569091 0.6184639 -0.2052447 0.5383806 -0.039140770 -0.12216171 -0.2841459 0.5059598 -0.3108725 -0.5683456 Canigou        3
4 0.9118693 -0.4069392 -0.6969241 0.6416924 -0.2924417 0.5608187 -0.022276169 -0.09450074 -0.3713938 0.6103618 -0.2886387 -0.5516278 Canigou        4
5 0.9472691 -0.2855528 -0.7323109 0.6582930 -0.3851661 0.5981639  0.018301826 -0.03539756 -0.4699465 0.6382341 -0.3066143 -0.6452738 Canigou        5
6 0.8656797 -0.4891864 -0.4827292 0.5690858 -0.1075195 0.5040311 -0.073575094 -0.15450012 -0.2592921 0.5053961 -0.3897712 -0.4876190 Canigou        6
```

```
head(test_p)
```

```
         Ce         Eu         Hf        La          Nb        Nd          Pr          Sm          U         Y         Yb         Cs          Region ObsExcel
1 0.8436152 -0.4371311 -0.6986892 0.6108806 -0.09165266 0.5678994 -0.05727135 -0.06257847 -0.2952401 0.5795778 -0.6791163 -0.2802939       4C SM2 T3      137
2 0.8296569 -0.4288913 -0.5931482 0.6062029 -0.10907806 0.5750894 -0.07167264 -0.03529184 -0.3124328 0.5658786 -0.6667615 -0.3595517       4C SM2 T3      138
3 0.8329848 -0.3990240 -0.6775466 0.6071688 -0.09857190 0.5797925 -0.07768562 -0.07166506 -0.3105109 0.6067131 -0.5676717 -0.4239835       4C SM2 T3      154
4 0.7901991 -0.5665613 -0.4622589 0.5676540 -0.09965719 0.5530605 -0.11750802 -0.09588293 -0.2758777 0.6311959 -0.5516726 -0.3726908 1L-SM10-2-L1ARR      157
5 0.7839590 -0.5226460 -0.4494427 0.5406361 -0.05813378 0.5306545 -0.13102718 -0.12889745 -0.2679789 0.5960488 -0.5075015 -0.3856709 1L-SM10-2-L1ARR      158
6 0.7394503 -0.5008568 -0.5018022 0.5192023 -0.07723161 0.4676393 -0.13846027 -0.10852438 -0.2335907 0.6489185 -0.3977027 -0.4170417 1L-SM10-2-L1ARR      159
```

```
data_main <- train_p    # data for building a tree
data_art <- test_p[5,]  # one artefact observation for analysis

out <- sifter(
  data_main = data_main, 
  class_col = 13, 
  internal_number_col = 14,
  labels_cex = 0.4,    # <-- changes the font size of the leaf labels
  mai_bottom = 0.8,    # <-- controls the free space on the bottom
  verbose = TRUE)
```

In this demonstration the height where the tree should be cut is specified (parameter `cutting_at_height`).

```
out <- sifter(
  data_main = data_main, 
  class_col = 13, 
  internal_number_col = 14,
  cutting_at_height = 4,
  labels_cex = 0.4, 
  mai_bottom = 0.8, 
  verbose = TRUE)
```

```
# The number of clusters at cutting_at_height = 4 
out$num_of_classes
```

```
[1] 2
```

Depending on the amount of data, the names in the leaves may be hard to see. In such a case, it is worth considering saving the drawing in a vector format, e.g. as pdf. In the example below, we first define the name of the directory and file with the resulting drawing. Then in the command `pdf(fig_name, width = 18, height = 8)`, by experimentally adjusting the values of the `width` and `height` parameters, we can obtain a drawing with the desired readability and quality.

```
fig_name <- paste("figs_sifter/my_tree_1.pdf", sep = "")
graphics.off()
pdf(fig_name, width = 18, height = 8)

out <- sifter(
  data_main = data_main, 
  class_col = 13, 
  internal_number_col = 14,
  labels_cex = 0.7, 
  mai_bottom = 1, 
  verbose = TRUE) 

dev.off()
```

In the last step, we need to load a script with some dedicated functions

```
source("funs.R")
```

# 6 Some notes related to data imputation

Back to top

The dataset used in the article (see `przeworsk.csv` file in the `data` directory) was originally partially incomplete. In our research missing data has been replaced by the **lowest regional values** (`lrv`). Such replacement procedure is known in literature as **data imputation**. In this chapter we use `przeworsk_NAs.csv` file (also included in the `data` directory) where missing values are not imputed yet. R package `mice` (Multivariate Imputation by Chained Equations) https://cran.r-project.org/web/packages/mice/index.html was used to demonstrate how the data imputation process can be carried out in practice. We used an advanced method called `pmm` (Predictive Mean Matching) and compare it with the `lrv` method used in the paper.

Back to top

## 6.1 Loading the original data

```
przeworsk_NAs <- read.csv(
  file = "data/przeworsk_NAs.csv", 
  dec = ".", 
  sep = ";", 
  header = TRUE, 
  stringsAsFactors = TRUE)
prz <- przeworsk_NAs[, c(9, 10, 11, 14, 7)]
col.names <-  c("Si", "Ca", "Al", "Mn", "Region")
names(prz) <- col.names
```

Several dozen sample items from the dataset are shown below and it is easy to see the `NA` values (Not Available / Missing Values).

```
prz[c(10:30, 230:235),]
```

```
         Si     Ca      Al     Mn Region
10   7.5719 0.2001  2.3025 1.4300    HCM
11  13.6154 0.7933      NA 0.1100    HCM
12  12.1150 0.7218      NA 3.1290    HCM
13  14.7138 0.3574      NA 0.8907    HCM
14  21.9351 0.4431      NA 0.7280    HCM
15  11.0634 0.6146  1.0692 0.5731    HCM
16  11.4420 1.1578  0.9898 0.2014    HCM
17  11.1335 1.8010  9.4745 1.2237    HCM
18  10.1987 1.3722 10.0355 0.5422    HCM
19  27.3429 1.4294  6.8280 0.1084    HCM
20  14.3071 1.5938  6.7009 0.2866    HCM
21  19.4251 2.6515  4.8484 0.1657    HCM
22       NA     NA      NA     NA    HCM
23       NA     NA      NA     NA    HCM
24  16.9199 1.5438  5.8752 0.1100    HCM
25  24.4918 6.0464  3.1546 1.3786    HCM
26       NA     NA      NA     NA    HCM
27  13.2742 1.1435  4.2344 0.5964    HCM
28  34.1202 1.1864  1.4715     NA    HCM
29  14.4193 3.3591  4.7849 0.7668    HCM
30  11.1849 1.2865  4.2185 3.6402    HCM
230  8.3431 0.4646  3.7898     NA    JUR
231  7.9925 0.0715  1.7202     NA    JUR
232  5.7958 2.9303  1.5032     NA    JUR
233 14.7979 1.7153  3.0646 4.9955    JUR
234  9.0535 1.0077  3.8639 0.6600    JUR
235 13.1386 0.9148  0.6669 1.3786    JUR
```

## 6.2 Visualisation of NA’s values

Back to top

In the `mice` R package there exists a very useful function `md.pattern` that can be used to quickly and graphically inspect the missing data patterns.

For example the missingness pattern in the upper left plot shows that in the `HCM` region there are 20 missing values in total: 8 for `Al`, 6 for `Mn` and 3 for `Si` and `Ca`. Moreover, there are 69 completely observed rows, 8 rows with 1 missing and 3 rows with 4 missings. Inspecting the missing data pattern is always useful (but may be difficult for datasets with many variables). It can give an indication on how much information is missing and how the missingness is distributed. The other three plots are analogous.

```
par(mfrow = c(2,2), pty="m")

data <- prz[which(prz$Region == 'HCM'),]
md.pattern(data[, 1:4])
text(1.7, 4.8, substitute(paste(bold('region: HCM'))))

data <- prz[which(prz$Region == 'JUR'),]
md.pattern(data[, 1:4])
text(1.7, 3.8, substitute(paste(bold('region: JUR'))))

data <- prz[which(prz$Region == 'MAS'),]
md.pattern(data[, 1:4])
text(1.7, 2.8, substitute(paste(bold('region: MAS'))))

data <- prz[which(prz$Region == 'SIL'),]
md.pattern(data[, 1:4])
text(1.7, 2.8, substitute(paste(bold('region: SIL'))))
```

## 6.3 Imputation and density visualization

Back to top

To demonstrate the results of the imputation, we will use a simple and very intuitive method of visually comparing the probability density plots of the raw data (i.e. those containing `NA` values) and the data after the missing values have been imputed.

The following 4 subsections contain the results of imputation of missing data for each region separately.

Comparing the plots in pairs for individual regions, it is easy to see that the results obtained using the `pmm` method are slightly closer to the results obtained with the dataset containing NA values than those obtained for the `lrv` method.

The greatest differences between the `pmm` and `lrv` methods are visible for the `MAS` region and `Al` element. However, it is easy to see that for this region the amount of missing data is the largest (as many as 17).

### 6.3.1 Make the imputation by regions

Back to top

```
data.HCM <- prz[which(prz$Region == 'HCM'),]
data.JUR <- prz[which(prz$Region == 'JUR'),]
data.MAS <- prz[which(prz$Region == 'MAS'),]
data.SIL <- prz[which(prz$Region == 'SIL'),]

out.HCM.pmm <- do_imputations(data.HCM, 'pmm')
out.HCM.lrv <- do_imputations(data.HCM, 'lrv')
out.JUR.pmm <- do_imputations(data.JUR, 'pmm')
out.JUR.lrv <- do_imputations(data.JUR, 'lrv')
out.MAS.pmm <- do_imputations(data.MAS, 'pmm')
out.MAS.lrv <- do_imputations(data.MAS, 'lrv')
out.SIL.pmm <- do_imputations(data.SIL, 'pmm')
out.SIL.lrv <- do_imputations(data.SIL, 'lrv')
```

### 6.3.2 `HCM` region results

Back to top

```
m <- paste("Method: ", out.HCM.pmm$method, sep = "")
figs <- ggarrange(
  out.HCM.pmm$ggplots.list[[1]], 
  out.HCM.pmm$ggplots.list[[2]], 
  out.HCM.pmm$ggplots.list[[3]], 
  out.HCM.pmm$ggplots.list[[4]]
)
annotate_figure(figs, top = text_grob(m, color = "red", face = "bold", size = 14))
```

```
m <- paste("Method: ", out.HCM.lrv$method, sep = "")
figs <- ggarrange(
  out.HCM.lrv$ggplots.list[[1]], 
  out.HCM.lrv$ggplots.list[[2]], 
  out.HCM.lrv$ggplots.list[[3]], 
  out.HCM.lrv$ggplots.list[[4]]
)
annotate_figure(figs, top = text_grob(m, color = "red", face = "bold", size = 14))
```

### 6.3.3 `JUR` region results

Back to top

```
m <- paste("Method: ", out.JUR.pmm$method, sep = "")
figs <- ggarrange(
  out.JUR.pmm$ggplots.list[[1]], 
  out.JUR.pmm$ggplots.list[[2]], 
  out.JUR.pmm$ggplots.list[[3]], 
  out.JUR.pmm$ggplots.list[[4]]
)
annotate_figure(figs, top = text_grob(m, color = "red", face = "bold", size = 14))
```

```
m <- paste("Method: ", out.JUR.lrv$method, sep = "")
figs <- ggarrange(
  out.JUR.lrv$ggplots.list[[1]], 
  out.JUR.lrv$ggplots.list[[2]], 
  out.JUR.lrv$ggplots.list[[3]], 
  out.JUR.lrv$ggplots.list[[4]]
)
annotate_figure(figs, top = text_grob(m, color = "red", face = "bold", size = 14))
```

### 6.3.4 `MAS` region results

Back to top

```
m <- paste("Method: ", out.MAS.pmm$method, sep = "")
figs <- ggarrange(
  out.MAS.pmm$ggplots.list[[1]], 
  out.MAS.pmm$ggplots.list[[2]], 
  out.MAS.pmm$ggplots.list[[3]], 
  out.MAS.pmm$ggplots.list[[4]]
)
annotate_figure(figs, top = text_grob(m, color = "red", face = "bold", size = 14))
```

```
m <- paste("Method: ", out.MAS.lrv$method, sep = "")
figs <- ggarrange(
  out.MAS.lrv$ggplots.list[[1]], 
  out.MAS.lrv$ggplots.list[[2]], 
  out.MAS.lrv$ggplots.list[[3]], 
  out.MAS.lrv$ggplots.list[[4]]
)
annotate_figure(figs, top = text_grob(m, color = "red", face = "bold", size = 14))
```

### 6.3.5 `SIL` region results

Back to top

```
m <- paste("Method: ", out.SIL.pmm$method, sep = "")
figs <- ggarrange(
  out.SIL.pmm$ggplots.list[[1]], 
  out.SIL.pmm$ggplots.list[[2]], 
  out.SIL.pmm$ggplots.list[[3]], 
  out.SIL.pmm$ggplots.list[[4]]
)
annotate_figure(figs, top = text_grob(m, color = "red", face = "bold", size = 14))
```

```
m <- paste("Method: ", out.SIL.lrv$method, sep = "")
figs <- ggarrange(
  out.SIL.lrv$ggplots.list[[1]], 
  out.SIL.lrv$ggplots.list[[2]], 
  out.SIL.lrv$ggplots.list[[3]], 
  out.SIL.lrv$ggplots.list[[4]]
)
annotate_figure(figs, top = text_grob(m, color = "red", face = "bold", size = 14))
```

## 6.4 Saving datasets after imputation

The following codes collect all the partial results and prepare the resulting files, where the missing data is replaced with the imputed data. These files are `przeworsk_pmm.csv` for the `pmm` method and `przeworsk_lrv.csv` for the `lrv` method. Both files are saved in the `data` directory.

```
przeworsk_pmm <- przeworsk_NAs
przeworsk_lrv <- przeworsk_NAs

rows.HCM <- as.numeric(rownames(data.HCM))
rows.JUR <- as.numeric(rownames(data.JUR))
rows.MAS <- as.numeric(rownames(data.MAS))
rows.SIL <- as.numeric(rownames(data.SIL))

# c(9, 10, 11, 14, 7)] - see 'Loading the original data (compositional)' section
przeworsk_pmm[rows.HCM, c(9, 10, 11, 14, 7)] <- out.HCM.pmm$data.imp
przeworsk_pmm[rows.JUR, c(9, 10, 11, 14, 7)] <- out.JUR.pmm$data.imp
przeworsk_pmm[rows.MAS, c(9, 10, 11, 14, 7)] <- out.MAS.pmm$data.imp
przeworsk_pmm[rows.SIL, c(9, 10, 11, 14, 7)] <- out.SIL.pmm$data.imp

przeworsk_lrv[rows.HCM, c(9, 10, 11, 14, 7)] <- out.HCM.lrv$data.imp
przeworsk_lrv[rows.JUR, c(9, 10, 11, 14, 7)] <- out.JUR.lrv$data.imp
przeworsk_lrv[rows.MAS, c(9, 10, 11, 14, 7)] <- out.MAS.lrv$data.imp
przeworsk_lrv[rows.SIL, c(9, 10, 11, 14, 7)] <- out.SIL.lrv$data.imp

# save numbers in fixed NOT in exponential notation
options(scipen = 10)

write.table(
  przeworsk_pmm, 
  file = "data/przeworsk_pmm.csv", 
  quote = FALSE,
  row.names = FALSE,
  sep = ";", 
  dec = ".",
  na = ""
)

write.table(
  przeworsk_lrv, 
  file = "data/przeworsk_lrv.csv", 
  quote = FALSE,
  row.names = FALSE,
  sep = ";", 
  dec = ".",
  na = ""
)
```

## 6.5 Inspecting the imputed datasets

Finally, one can compare the original data shown in Loading the original data Section with the below ones.

```
przeworsk_lrv[c(10:30, 230:235), c(9, 10, 11, 14, 7)]
```

```
         Si     Ca      Al     Mn Region
10   7.5719 0.2001  2.3025 1.4300    HCM
11  13.6154 0.7933  0.2911 0.1100    HCM
12  12.1150 0.7218  0.2911 3.1290    HCM
13  14.7138 0.3574  0.2911 0.8907    HCM
14  21.9351 0.4431  0.2911 0.7280    HCM
15  11.0634 0.6146  1.0692 0.5731    HCM
16  11.4420 1.1578  0.9898 0.2014    HCM
17  11.1335 1.8010  9.4745 1.2237    HCM
18  10.1987 1.3722 10.0355 0.5422    HCM
19  27.3429 1.4294  6.8280 0.1084    HCM
20  14.3071 1.5938  6.7009 0.2866    HCM
21  19.4251 2.6515  4.8484 0.1657    HCM
22   0.7104 0.0500  0.2911 0.0008    HCM
23   0.7104 0.0500  0.2911 0.0008    HCM
24  16.9199 1.5438  5.8752 0.1100    HCM
25  24.4918 6.0464  3.1546 1.3786    HCM
26   0.7104 0.0500  0.2911 0.0008    HCM
27  13.2742 1.1435  4.2344 0.5964    HCM
28  34.1202 1.1864  1.4715 0.0008    HCM
29  14.4193 3.3591  4.7849 0.7668    HCM
30  11.1849 1.2865  4.2185 3.6402    HCM
230  8.3431 0.4646  3.7898 0.2246    JUR
231  7.9925 0.0715  1.7202 0.2246    JUR
232  5.7958 2.9303  1.5032 0.2246    JUR
233 14.7979 1.7153  3.0646 4.9955    JUR
234  9.0535 1.0077  3.8639 0.6600    JUR
235 13.1386 0.9148  0.6669 1.3786    JUR
```

```
przeworsk_pmm[c(10:30, 230:235), c(9, 10, 11, 14, 7)]
```

```
         Si     Ca      Al     Mn Region
10   7.5719 0.2001  2.3025 1.4300    HCM
11  13.6154 0.7933  5.7694 0.1100    HCM
12  12.1150 0.7218  1.0110 3.1290    HCM
13  14.7138 0.3574  2.5406 0.8907    HCM
14  21.9351 0.4431  3.0170 0.7280    HCM
15  11.0634 0.6146  1.0692 0.5731    HCM
16  11.4420 1.1578  0.9898 0.2014    HCM
17  11.1335 1.8010  9.4745 1.2237    HCM
18  10.1987 1.3722 10.0355 0.5422    HCM
19  27.3429 1.4294  6.8280 0.1084    HCM
20  14.3071 1.5938  6.7009 0.2866    HCM
21  19.4251 2.6515  4.8484 0.1657    HCM
22  11.3765 6.0464  1.0321 0.7668    HCM
23  12.6198 0.7790  2.5248 0.8287    HCM
24  16.9199 1.5438  5.8752 0.1100    HCM
25  24.4918 6.0464  3.1546 1.3786    HCM
26  19.9720 0.4431  4.8484 0.1084    HCM
27  13.2742 1.1435  4.2344 0.5964    HCM
28  34.1202 1.1864  1.4715 0.1084    HCM
29  14.4193 3.3591  4.7849 0.7668    HCM
30  11.1849 1.2865  4.2185 3.6402    HCM
230  8.3431 0.4646  3.7898 0.6600    JUR
231  7.9925 0.0715  1.7202 0.7300    JUR
232  5.7958 2.9303  1.5032 0.2246    JUR
233 14.7979 1.7153  3.0646 4.9955    JUR
234  9.0535 1.0077  3.8639 0.6600    JUR
235 13.1386 0.9148  0.6669 1.3786    JUR
```
