# Supplementary material for: Old wine into new wineskins? “Legacy data” in research on Roman Period East Germanic iron smelting
Source: PLoS One. 2023 Oct 19;18(10):e0289771. doi: 10.1371/journal.pone.0289771 (PMC10586651; doi:10.1371/journal.pone.0289771)
Supplement: S1 File — (ZIP) [file my_tree_1.pdf › S4 R Materials/figs_sifter/my_tree_1.pdf]

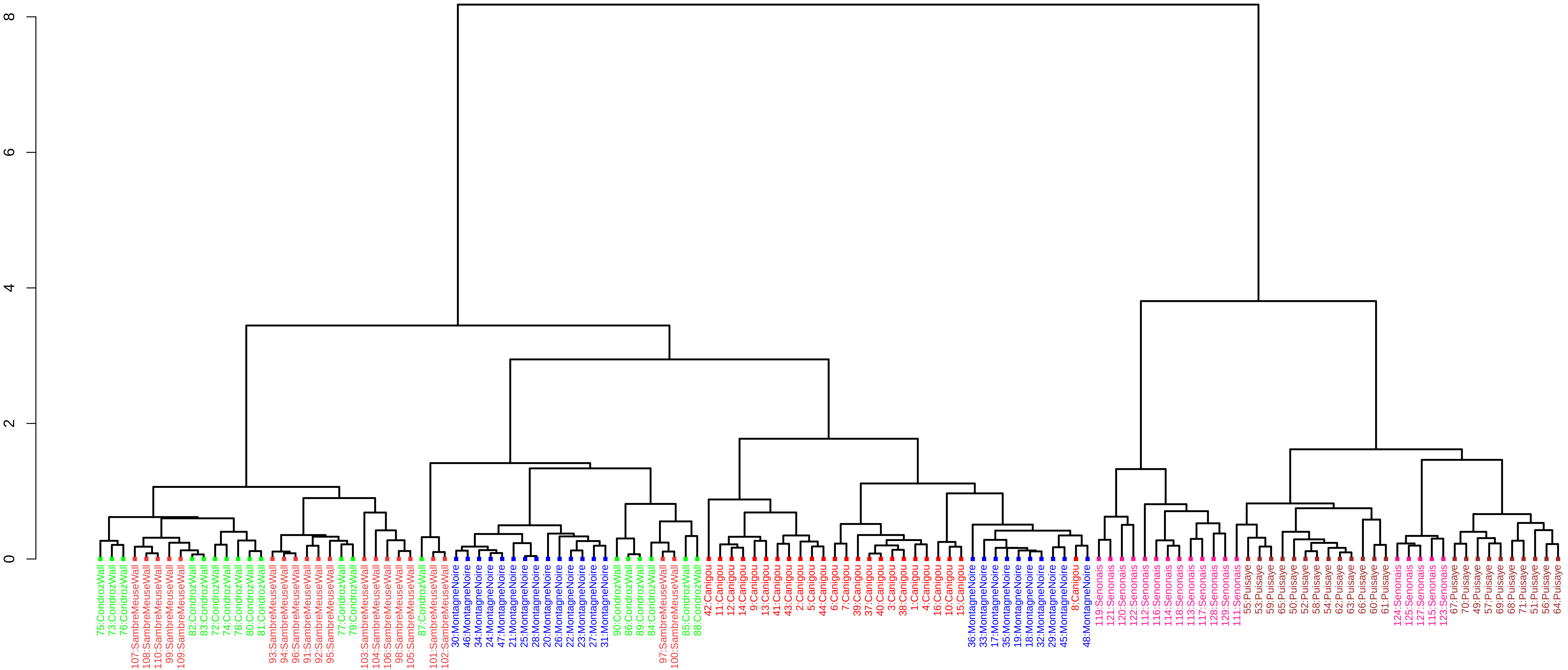
8
6
4
2
0
75:CondrozWall
73:CondrozWall
76:CondrozWall
107:SambreMeuseWall
108:SambreMeuseWall
110:SambreMeuseWall
99:SambreMeuseWall
109:SambreMeuseWall
82:CondrozWall
83:CondrozWall
72:CondrozWall
74:CondrozWall
78:CondrozWall
80:CondrozWall
81:CondrozWall
93:SambreMeuseWall
94:SambreMeuseWall
96:SambreMeuseWall
91:SambreMeuseWall
92:SambreMeuseWall
95:SambreMeuseWall
77:CondrozWall
79:CondrozWall
103:SambreMeuseWall
104:SambreMeuseWall
106:SambreMeuseWall
98:SambreMeuseWall
105:SambreMeuseWall
87:CondrozWall
101:SambreMeuseWall
102:SambreMeuseWall
30:MontagneNoire
46:MontagneNoire
34:MontagneNoire
24:MontagneNoire
47:MontagneNoire
21:MontagneNoire
25:MontagneNoire
28:MontagneNoire
20:MontagneNoire
26:MontagneNoire
22:MontagneNoire
23:MontagneNoire
27:MontagneNoire
31:MontagneNoire
90:CondrozWall
86:CondrozWall
89:CondrozWall
84:CondrozWall
97:SambreMeuseWall
100:SambreMeuseWall
85:CondrozWall
88:CondrozWall
42:Canigou
11:Canigou
12:Canigou
14:Canigou
9:Canigou
13:Canigou
41:Canigou
43:Canigou
2:Canigou
5:Canigou
44:Canigou
6:Canigou
7:Canigou
39:Canigou
37:Canigou
40:Canigou
3:Canigou
38:Canigou
1:Canigou
4:Canigou
16:Canigou
10:Canigou
15:Canigou
36:MontagneNoire
33:MontagneNoire
17:MontagneNoire
35:MontagneNoire
19:MontagneNoire
18:MontagneNoire
32:MontagneNoire
29:MontagneNoire
45:MontagneNoire
8:Canigou
48:MontagneNoire
119:Senonais
121:Senonais
120:Senonais
122:Senonais
112:Senonais
116:Senonais
114:Senonais
118:Senonais
113:Senonais
117:Senonais
128:Senonais
129:Senonais
111:Senonais
55:Puisaye
53:Puisaye
59:Puisaye
65:Puisaye
50:Puisaye
52:Puisaye
58:Puisaye
54:Puisaye
62:Puisaye
63:Puisaye
66:Puisaye
60:Puisaye
61:Puisaye
124:Senonais
125:Senonais
127:Senonais
115:Senonais
123:Senonais
67:Puisaye
70:Puisaye
49:Puisaye
57:Puisaye
69:Puisaye
68:Puisaye
71:Puisaye
51:Puisaye
56:Puisaye
64:Puisaye
